# Effectiveness and safety of negative pressure wound therapy in patients with deep sternal wound infection: a systematic review and meta-analysis

**DOI:** 10.1097/JS9.0000000000002138

**Published:** 2024-11-14

**Authors:** Yen-Ting Liu, Shih-Han Lin, Chi Peng, Ren-Wen Huang, Cheng-Hung Lin, Chung-Chen Hsu, Shih-Heng Chen, Yu-Te Lin, Che-Hsiung Lee

**Affiliations:** a Department of Plastic and Reconstructive Surgery, Chang Gung Memorial Hospital, Taoyuan, Taiwan and College of Medical, Chang Gung University, Taoyuan, Taiwan; b School of Medicine, Chang Gung University, Taoyuan, Taiwan; c Center for Vascularized Composite Allotransplantation, Chang Gung Memorial Hospital, Taoyuan, Taiwan

**Keywords:** chest wall reconstruction, deep sternal wound infection, meta-analysis, negative pressure wound therapy, systematic review, vacuum-assisted closure

## Abstract

**Background:**

Deep sternal wound infection (DSWI) is a severe and life-threatening complication following cardiovascular surgery. Negative pressure wound therapy (NPWT) has emerged as a promising therapeutic bridging option for DSWI. In this systematic review and meta-analysis, the authors aimed to evaluate the impact of NPWT on clinical outcomes in patients with DSWI.

**Material and Methods:**

A comprehensive literature search was conducted according to the PRISMA guideline in electronic databases, including PubMed, Embase, and Cochrane Library. Data extraction was performed independently by two reviewers, and risk of bias was assessed by ROBINS-I tool. The primary outcomes assessed were mortality rate and reinfection rate. The secondary outcomes assessed were length of hospital stay and ICU stay.

**Results:**

In this systematic review identified a total of 36 studies, comprising 3681 patients with DSWI who received treatment. The meta-analysis revealed that NPWT was associated with a significant reduction in mortality rate (RR 0.46, 95% CI: 0.35–0.61, *P*<0.000001) and reinfection rate (RR 0.43, 95% CI: 0.25–0.74, *P*=0.002) compared to conventional wound management. Furthermore, pooling of these studies showed significant difference between the NPWT and conventional treatment groups in length of hospital stay (mean difference: −4.49, 95% CI: −8.14 to −0.83; *P*=0.02) and length of ICU stay (mean difference: −1.11, 95% CI: −2.18 to −0.04; *P*=0.04).

**Conclusion:**

This systematic review and meta-analysis provide evidence that NPWT is superior to conventional treatment for patients with DSWI following cardiovascular surgery.

## Introduction

Highlights

- Significant reduction in mortality and reinfection rates: The meta-analysis demonstrated that Negative Pressure Wound Therapy (NPWT) significantly reduces mortality and reinfection rates compared to conventional wound management.
- Decreased hospital and ICU stay: NPWT was associated with a significantly shorter length of hospital stay and ICU stay.
- Safety and complications: While NPWT is generally safe, the most frequently reported complications include right ventricular rupture and major bleeding events. These complications highlight the importance of adhering to guidelines and using protective measures during therapy to mitigate risks.

Deep sternal wound infection (DSWI), also known as mediastinitis, which may be commonly caused by different etiologies including open cardiac surgery or descending necrotizing mediastinitis (DNM) arising from Ludwig’s angina^1,2^. DSWI represents a grave complication postopen cardiovascular surgery, with an incidence rate of 1–4% as reported in the literature^3,4^. The progression of DSWI can lead to devastating consequences, including osteomyelitis, sepsis, ventricular rupture, and graft infection, culminating in a mortality rate up to 35%^5^. Such outcomes underscore the necessity for more assertive treatment strategies to mitigate these severe consequences.

Managing sternal infections presents a significant clinical challenge. Traditional treatment methods, including debridement, antibiotics, irrigation, open dressing, and immediate flap reconstruction, have not yielded satisfactory results. However, the advent of negative pressure wound therapy (NPWT) in the late 1990s marked a significant advancement, demonstrating efficacy in both chronic and acute wound care by diminishing local edema, facilitating debris removal, exerting microdeformation forces, and expediting granulation tissue development^6^. NPWT has since gained recognition as an effective intervention for DSWI, serving as a bridge to sternal reconstruction or as a means for definitive closure^7,8^.

Emerging studies after the introduction of NPWT have consistently evidenced its clinical benefits in DSWI management—namely, a reduction in dressing changes, the necessity for flap reconstruction, and hospital stay durations^9^. Moreover, NPWT has been instrumental in enhancing sternal stabilization, thereby promoting pain alleviation, enabling earlier extubation, and facilitating patient mobilization^10^. A previous meta-analysis^11,12^ highlighted a significant decrease in hospitalization duration and mortality rate among patients managed with NPWT compared to conventional treatment modalities.

Over the past decade, a proliferation of studies has broadened the understanding of the impact, efficacy, and safety of NPWT in the context of DSWI. In addition, the innovations in NPWT equipment, such as the introduction of NPWT instillation and dwell, improvements in products like reticulated open-cell foams (ROCF) led to the progress in the treatment for DSWI. However, the latest guidelines for the treatment of deep sternal wound infections, published by AATS in 2016^13^ and EACTS^14^ in 2017, only include literatures up to 2015. There was no updated study to reach the consensus on the therapeutic approach of NPWT since then. Therefore, in this article, we focus on conducting a systematic review and meta-analysis to compare the impact of NPWT and conventional treatment on the outcomes of patients who developed DSWI following cardiac surgery. We aim to update the latest evidence on the application of NPWT including the effectiveness, safety, clinical role, and implementation details for DSWI.

## Material and methods

This systematic review adheres to the Preferred Reporting Items for Systematic Reviews and Meta-Analyses (PRISMA) guidelines^15^ (Supplemental Digital Content 1, http://links.lww.com/JS9/D530) and Assessing the methodological quality of systematic review 2 (AMSTAR2) guidelines^16^ (Supplemental Digital Content 2, http://links.lww.com/JS9/D531). This research has been registered in PROSPERO (registration ID: CRD42024470569) and Research Registry (UIN: reviewregistry1894, https://www.researchregistry.com).

### Diagnosis and definition of DSWI

DSWI diagnosis aligns with the Centers for Disease Control and Prevention criteria^17^, requiring at least one of the following: positive mediastinal tissue or fluid cultures, evidence of mediastinitis via surgical observation or histopathology, or signs such as fever, chest pain, or sternal instability, accompanied by purulent mediastinal discharge, positive blood or mediastinal discharge cultures, or mediastinal widening on X-ray.

### Definition of NPWT and conventional wound treatment

NPWT, as known as vacuum-assisted closure (VAC), was defined as wound dressing system that applied subatmospheric pressure continuously or intermittently to the system, which provides a negative pressure to wound surface and promotes wound healing.

The definition of conventional wound treatment was listed in Table 1 from each of the included study. These treatments referred to debridement, close irrigation, open irrigation, open packing, reconstruction with PM flap, omental flap, or other flaps.

**Table 1:** Study characteristic.

First author/year	Study design, study years, country	No. of participant (NPWT vs conventional)	NPWT indication (Bridge/Definite/Nondetermined)	Conventional treatment	Mortality (%) (NPWT vs conventional)	Reinfection rate (%) (NPWT vs conventional)	Length of hospital stay (days) (NPWT vs conventional)	Length of ICU stay (days) (NPWT vs conventional)
Berg 2000	Retrospective cohort; 1989–1997; Netherlands	60 (31 vs 29)	Definite	Continuous irrigation	6.45 vs 6.9	N/A	42 (±26) vs 55 (±22)	N/A
Doss 2002	Retrospective cohort; 1998–2000; Germany	42 (20 vs 22)	Definite	Re-exploration, removal of wire, debridement, irrigation, open drainage	5 vs 4.55	N/A	27.2 (±6.5) vs 33 (±11)	N/A
Fleck TM 2002	retrospective cohort; no mention; Austria	11 (6 vs 5)	Nondetermined	Primary wound closure with rewiring of the sternum and PMMF closure	0 vs 0	N/A	31.17 (±8.47) vs 29.4 (±13.5)	1.75 (±0.97) vs 12.25 (±6.55)
Domkowski 2003	Retrospective cohort; 1997–2002; United Kingdom	102 (96 vs 6)	Nondetermined	Multiple dressing change	4.17 vs 0	N/A	27 (±12) Vs N/A	N/A
Fuchs 2005	Retrospective cohort; 1998–2003; Germany	68 (35 vs 33)	Definite	Irrigation, wound drainage, open packing, delayed closure	2.86 vs 12.1	N/A	25 (±13.1) vs 34 (±23.99)	N/A
Immer 2005	Retrospective cohort; 1998–2003; Switzerland	55 (38 vs 17)	Nondetermined	Sternal excision, primary musculocutaneous flap	2.63 vs 11.76	N/A	70.3 (±45.27) vs 70.7 (±28.5)	2.25 (±2.11) vs 4.8 (±7.8)
Segers 2005	Retrospective cohort; 1992–2003; Netherlands	63 (29 vs 34)	Nondetermined	Irrigation and close drainage	31.03 vs 26.47	27.58 vs 52.94	46.1 (±16) vs 35.7 (±38.75)	N/A
Sjogren 2005	Retrospective cohort; 1994–2003; Sweden	101 (61 vs 40)	Definite	Rewiring/open dressing/closed irrigation/pectoralis flap/omentoplasty	0 vs 15	6.56 vs 4.76	25 (±17) vs 25 (±20)	16 (±10)vs 17 (±16)
Chen Y 2008	Retrospective cohort; 2001–2006; Australia	31 (26 vs 5)	Nondetermined	Delay closure and pectoralis major advancement flap	23.08 vs 20	N/A	39.8 (±24) vs N/A	N/A
Eyileten 2009	Retrospective cohort; 2000–2007; Turkey	65 (33 vs 32)	Nondetermined	Early bilateral PMMF and continuous irrigation	3.03 vs 6.25	3.03 vs 0	25.9 (±20.2) vs 33.7 (±16.5)	N/A
Baillot R 2010	Retrospective cohort; 1992-2007; Canada	267 (125 vs 142)	Bridge	Primary closure and irrigation (*N*=37), debridement/drainage, open packing followed by PMMFs (*N*=81)	4.8 vs 14.08	N/A	N/A	N/A
De Feo 2010	Retrospective cohort; 1979–2009; Italy	69 (39 vs 30)	Bridge	Close irrigation w/ antibiotics	0 vs 3.33	0 vs 23.33	26.6 (±8.4) vs 36.4 (±3.2)	13.5 (±3.2) vs 21.2 (±16.4)
Petzina 2010	Retrospective cohort; 2004-2009; Germany	118 (69 vs 49)	Nondetermined	Debridement, drainage, and irrigation, omental flap	5.8 vs 24.29	2.9 vs 18.37	43 (±15.4) vs 51 (±26.7)	N/A
Assmann 2011	Retrospective cohort; 2004-2008; Germany	154 (82 vs 72)	Nondetermined	Primary rewiring and disinfectant irrigation after DSWI	14.63 vs 26.39	N/A	45.6 (±18.5) vs 55.2 (±23.6)	2.1 (±1.2) vs 3.9 (±1.7)
Marisa De Feo 2011	Retrospective cohort; 1995–2010; US	157 (74 vs 83)	Nondetermined	Primary wound reopening, closed-chest irrigation without rewiring, topical application of granulated sugar, and final plastic reconstruction with pectoral muscle flap	1.35 vs 3.61	1.35 vs 16.87	27.3 (±9) vs 30.5 (±3)	1.8 (±1.1) vs 3.2 (±2.9)
De Feo 2011	Retrospective cohort; 1979–2009; Italy	200 (55 vs 145)	Bridge	Non-VAC: Group A (anti) and Group B (debridement, close irrigation, open dressing) (total *n*=145)	1.82 vs 14.48	N/A	27.3 (±9) vs 36.27 (±9.2)	N/A
Kobayashi 2011	Retrospective cohort; 2001–2007; Japan	16 (9 vs 7)	Bridge	total sternectomy and primary wound closure + omental/PM flap; sternal preservation and delayed closure with omental/PM flap	0 vs 14.29	0 vs 57.14	63.4 (±54.1) vs 120 (±31.8)	N/A
Morisaki 2011	Retrospective cohort; 1991-2010; Japan	59 (8 vs 51)	Nondetermined	Simple closure or tissue flap reconstruction after debridement	0 vs 27.45	N/A	94 (±80.4) vs 57.5 (±34.7)	N/A
Deniz 2012	Retrospective cohort; 2000–2011; Turkey	90 (47 vs 43)	Bridge	Surgical revision and debridement, open dressing, closed irrigation, pectoral flap	8.51 vs 23.26	2.13 vs 4.65	26 (±8) vs 31 (±9)	N/A
Rodriguez CB 2012	Retrospective cohort; 1999-2008; Portugal	159 (105 vs 54)	Nondetermined	Direct wound closure (debride, wound irrigation, rewiring)	1.9 vs 5.56	N/A	21.1 (±16.4) vs 13.3 (±12.1)	4.1 (±5) vs 3.2 (±2.3)
Simek 2012	Retrospective cohort; 2002-2007; Czeck Republic	62 (34 vs 28)	Nondetermined		5.88 vs 28.57	N/A	N/A	N/A
Steingrimsson 2012	Retrospective cohort; 2000–2010; Iceland	43 (20 vs 23)	Nondetermined	Open and/or close irrigation	0 vs 4.35	5 vs 34.78	47 (±17) vs 48 (±23)	3.7 (±7.6) vs 4.3 (±7.4)
Vos RJ 2012	Retrospective cohort; 2000–2010; Netherlands	113 (89 vs 24)	Nondetermined	Open packing	12.36 vs 41.67	N/A	74 (±61) vs 69 (±62)	6.8 (±14.4) vs 18.5 (±21)
Vos 2012	Retrospective cohort; 2000–2011; Netherlands	132 (89 vs 43)	Nondetermined	Close drainage with Redon catheter	12.36 vs 13.95	14.61 vs 6.98	7(±61) vs 4(±38)	6.(14.4) vs 4.(±10.1)
Fleck T 2014	Retrospective cohort; 1995–2011; Austria	524 (326 vs 198)	Nondetermined	Surgical debridement, irrigation, open packing, and muscle flap	3.68 vs 7.58	7.67 vs 23.74	22 (±9) vs N/A	N/A
Risnes I 2014	dynamic cohort; 1997-2010; Norway	130 (64 vs 66)	Nondetermined	Traditional close drainage with irrigation	3.13 vs 0	6.25 vs 21.21	N/A	N/A
Yumun G 2014	Retrospective cohort; 2008–2013; Turkey	58 (39 vs 19)	Definite	Primary surgical closure	15.38 vs 42.11	N/A	N/A	N/A
Morisaki 2016	Retrospective cohort; 1991–2015; Japan	44 (22 vs 22)	Nondetermined	Open daily irrigation	4.55 vs 36.36	N/A	N/A	8.8 (±16.1) vs 6.8 (±7.9)
Pan T 2020	Retrospective cohort; 2014–2018; China	132 (66 vs 66)	Nondetermined	Bilateral PMMF	13.64 vs 6.06	16.67 vs 9.09	33 (±18.94) vs 16.67 (±6.82)	3.67 (±1.52) vs 3.67 (±0.76)
Hämäläinen 2021	Retrospective cohort; 2007–2016; Finland	129 (55 vs 74)	Nondetermined	Revision and closure, revision, and continuous irrigation	3.64 vs 8.11	N/A	18 (±7.41) vs 38 (±17.78)	2 vs 1
Myllykangas 2021	Retrospective cohort; 2006–2018; Finland	115 (55 vs 60)	Nondetermined	Flap reconstruction without prior NPWT	14.55 vs 0	N/A	36.8 (±28.1) vs 25.6 (±38.7)	10.5 (±13.4) vs 6.9 (±17.7)
Banjanovic 2022	Retrospective cohort; 2015-2020; Bosnia	15 (8 vs 7)	Bridge	Surgical debridement, sternum fixation, and retrosternal irrigation.	28.57 vs 37.5	N/A	38.17 (±28.65) vs 23.5 (±13.15)	N/A
Gegouskov 2022	Retrospective cohort; 2010-2021; Bulgaria	77 (32 vs 45)	Nondetermined	Wound revision with primary closure, continuous wound irrigation, and open treatment with secondary closure	3.125 vs 0.2	6.25 vs 26.67	8 (±2.38) vs 16 (±4.25)	N/A
Myllykangas 2022	Retrospective cohort; 2006-2020; Finland.	58 (48 vs 10)	Bridge	PMMF	N/A	N/A	12.8 (±8.6) vs 22.1 (±42.1)	4.6 (±7.5) vs 8 (±19.67)
Akbayrak H 2023	Retrospective cohort; 2001–2013; Turkiye	114 (52 vs 62)	Nondetermined	Open packing and sternum rewiring or PMMF	5.77 vs 20.97	N/A	27.24 (±6.1) vs 76.11 (±42.74)	N/A
Saltarocchi S 2023	Retrospective cohort; 2012–2020; Italy.	34 (19 vs 15)	Bridge	Catheter irrigation and subsequent sternal closure or PMMF	0 vs 26.67	N/A	N/A	N/A

### Literature search

We systematically searched PubMed, Embase, and the Cochrane Library up to 1st December 2023, for comparative studies on NPWT and conventional for DSWI treatments. The search was confined to studies published in English. The following search terms were used with Boolean operators AND, OR, and NOT: ‘deep sternal wound infection’ or ‘mediastinitis’ or ‘osteomyelitis’, ‘negative pressure wound therapy’ or ‘vacuum-assisted closure’, ‘mortality rate’ or ‘reinfection rate’ or ‘length of hospital stay’. Related Medical Subject Headings (MeSH) were also searched. The comprehensive search strategy is detailed in Supplemental Digital Content 3, http://links.lww.com/JS9/D532 and Supplemental Digital Content 4, http://links.lww.com/JS9/D533. All references of included studies were reviewed to broaden the search for potentially eligible studies.

### Inclusion and exclusion criteria

**Figure 1:**
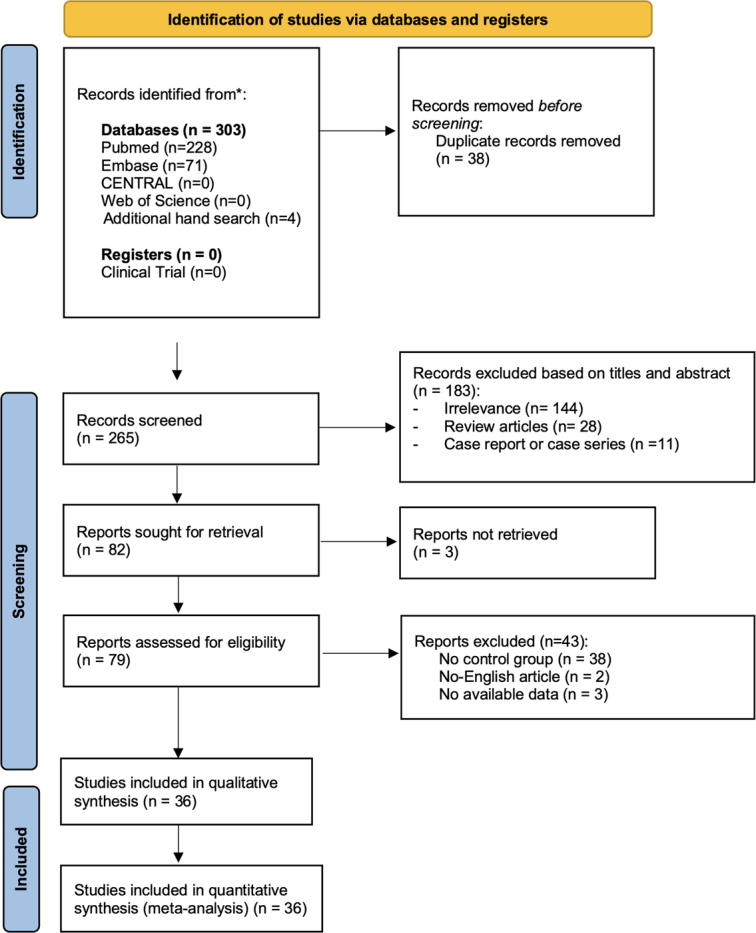
A figure of the Preferred Reporting Items for Systematic Reviews and Meta-Analyses diagram (PRISMA) flowchart.

We considered retrospective, cohort, nonrandomized controlled trials (non-RCTs), and randomized controlled trials (RCTs) that compared NPWT and conventional treatment outcomes for DSWI. We included articles involving patients who developed DSWI following open-heart surgery and articles where DSWI were caused by other reasons were excluded. Exclusion criteria encompassed patients with only superficial sternal wound infections or those under 18 years of age. The incisional NPWT for prevention of surgical wound infection is excluded from our study. Additional studies were identified by manually searching the bibliographies of eligible RCTs and reviews. We included articles in all language and non-English literature was translated for thorough evaluation. All articles that fulfilled the inclusion criteria were retrieved for full-text evaluation. The eligibility criteria was summarized in Table 2 and the inclusion and exclusion process were presented in Figure 1.

**Table 2:** Eligibility criteria.

Category	Criteria
Patient	Adults with DSWI following open cardiac surgery
Intervention	NPWT of any form applied to DSWI with intention to acceleration wound healing and staged reconstruction
Control	Conventional treatment applied to DSWI including close irrigation, open irrigation, open packing, or immediate flap reconstruction
Outcomes	Mortality, reinfection rate, length of hospital stays, length of ICU stays, complication rate
Study design	Randomized controlled trials, retrospective cohort studies, prospective cohort studies; exclusion of case series, case reports, reviews, and any study without a control group
Publication type	Clinical journal articles and abstracts
Language	No limitation

DSWI, deep sternal wound infection; NPWT, negative pressure wound therapy.

### Data extraction

**Figure 2:**
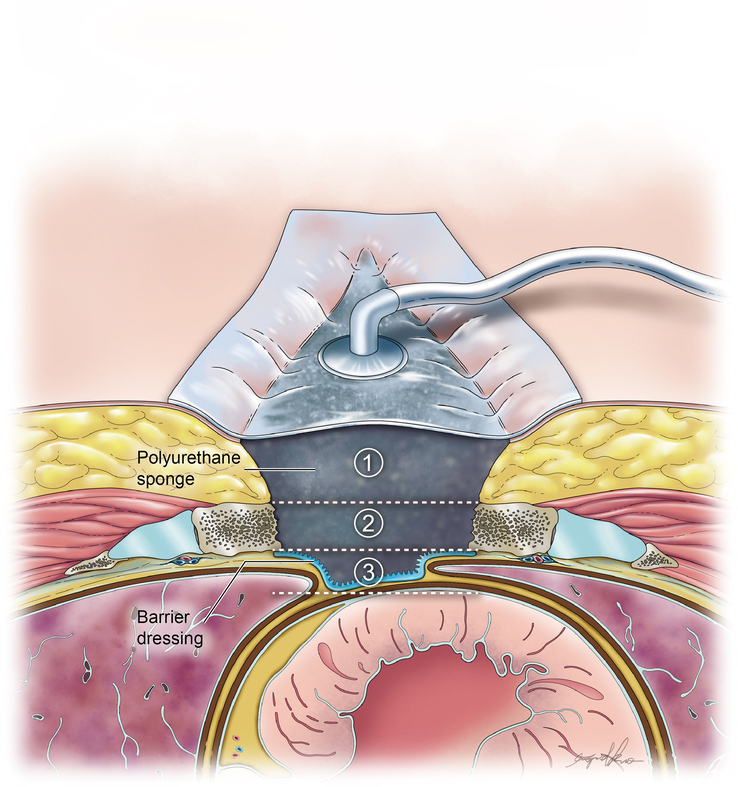
Illustration of NPWT application in the treatment of DSWI. The depth of NPWT application can be classified into three categories: (1) subcutaneous, (2) between the sternum edge, and (3) substernum. A barrier dressing is applied to protect vascular graft, cardiac tissue, and other vital organs from direct contact with the NPWT device.

Data was meticulously extracted by two independent reviewers (Y.T.L. and S.H.L.), with discrepancies resolved through consultation with a third reviewer (C.H.L.). Collected data was listed in Table 3 included study characteristics, conventional treatment modalities, primary outcomes (mortality and reinfection rates), secondary outcomes (hospital and ICU stay lengths), NPWT information, details of applications, and complications. The authors were contacted for missing details. The details of application were summarized in Table 4 and illustrated in Figure 2.

**Table 3:** Data extraction.

Category	Data
Publication details	Title, first author, publication year, country
Study characteristics	Study design, number of subjects (NPWT/conventional treatment)
Intervention	Form of NPWT, manufacturer, NPWT setting, duration of intervention
Outcomes	Mortality, reinfection rate, length of hospital stays, length of ICU stays, complication rate

NPWT, negative pressure wound therapy.

**Table 4:** Summary of NPWT application including visceral organ exposure, depth of application, and DSWI classification.

First author/year	Visceral organ exposures	Depth of application	Details of application	El Oakley classifications						
				I	II	IIIa	IIIb	IVa	IVb	V
Berg 2000	Yes	Substernum	Catheters are placed to the left and right, underneath and above the heart. Redon catheters are brought in beneath the subcutis. The subcutis is closed with absorbable dexon suture	N/A						
Doss 2002	N/A	N/A	Polyurethane foam was tailored to fit the size of the tissue defect	N/A						
Fleck TM 2002	Yes	Substernum	Pericardium was closed to ensure no adherence to the surface of the heart. Sponges were cut and fitted into the sternal wound proximally and distally	N/A						
Domkowski 2003	N/A	N/A	Deep sternal infection requiring removal of all wires. An additional vascular flap procedure was required	N/A						
Fuchs 2005	Yes	Substernum	Polyurethane VAC sponge was fitted into the wound substernally. Second sponge was placed between the sternal edges	0	9	12	10	0	0	4
Immer 2005	N/A	N/A	Debridement and exchange was performed every 48–72 h	0	38	0	0	0	0	0
Segers 2005	Yes	Substernum	One layer of foam was positioned underneath the sternum. A strip of foam was placed into the sternal defect itself. The most superficial layer of foam should rise to 4 cm above skin level	4	2	3	9	9	0	2
Sjogren 2005	Yes	Between the sternal edge	Paraffin gauze dressing were placed at the bottom of the wound covering. Isolating visible parts of vital organs and grafts from the sternal edges. Seal the gap between the bone edges	12	7	13	26	1	0	2
Chen Y 2008	No	Between the sternal edge	Polyurethane foam dressing placed between the sternal edges	N/A						
Eyileten 2009	No	Subcutaneous	Polyurethane foam was placed below the skin edges. One sternal wire was left. Looping in close proximity to the anterior surface of the heart	N/A						
Baillot R 2010	N/A	N/A	Delayed sternal osteosynthesis. Horizontal titanium plate fixation covered with PMF flaps	N/A						
De Feo 2010	Yes	Between the sternal edge	Paraffin gauze dressing were placed on the heart. Polyurethane foam dressing was trimmed to fit between the sternal edges	N/A						
Petzina 2010	Yes	Between the sternal edge	The surface of the right ventricle is protected by layers of paraffin gauze. The first layer of polyurethane foam is placed between the sternal edges	N/A						
Assmann 2011	N/A	N/A	N/A	N/A						
M. De Feo 2011	Yes	Between the sternal edge	Paraffin gauze dressing were placed on the heart. Polyurethane foam dressing was trimmed to fit between the sternal edges	11	5	19	34	4	0	1
De Feo 2011	Yes	Between the sternal edge	Paraffin gauze dressing were placed on the heart. Polyurethane foam dressing was trimmed to fit between the sternal edges	N/A						
Kobayashi 2011	Yes	Substernum	Polyurethane foam sponge was cut and fitted into the mediastinal space	0	9	0	0	0	0	0
Morisaki 2011	Yes	Substernum	Polyurethane sponges were mounted in the mediastinum sternum. Prevent injury to the heart	N/A						
Deniz 2012	N/A	N/A	Placing polyurethane foam with an open pore structure of 400–600 μm	5	2	17	22	1	0	0
Rodriguez CB 2012	N/A	N/A	N/A	N/A						
Simek 2012	N/A	N/A	N/A	N/A						
Steingrimsson 2012	Yes	Substernum	Paraffin gauze dressing was placed at the bottom of the wound. Polyurethane foam was placed in the wound in two layers	N/A						
Vos RJ 2012	Yes	Between the sternal edge	A polyvinyl alcohol dressing placed substernal to protect vital structures. Sponge was placed between the sternal edges and the subcutaneous layer	12	32	35	0	2	0	7
Vos 2012	Yes	Between the sternal edge	A polyvinyl alcohol dressing placed substernal to protect vital structures. Sponge was placed between the sternal edges and the subcutaneous layer	12	32	35	0	2	0	7
Fleck T 2014	Yes	Between the sternal edge	Nonadherent foam dressing was applied to avoid contact with the heart. VAC sponge was cut and fitted between the sternal edges. Prevent shear forces between the bony edges and the right ventricle	2	1	51	6	13	0	2
Risnes I 2014	Yes	between the sternal edge	Free the sternum from the right ventricle. Paraffin gauze was placed between the sternum and right ventricle. The first layer was placed between the sternal edges onto the heart	N/A						
Yumun G 2014	N/A	N/A	N/A	N/A						
Morisaki 2016	Yes	substernum	Polyurethane sponges were mounted in the mediastinum sternum. Prevent injury to the heart	1	7	3	4	2	0	5
Pan T 2020	N/A	N/A	N/A	100	218	0	0	0	0	0
Hämäläinen E 2021	N/A	N/A	N/A	N/A						
Myllykangas 2021	N/A	N/A	N/A	2	10	20	20	0	0	3
Banjanovic B 2022	N/A	N/A	N/A	N/A						
Gegouskov V 2022	Yes	Between the sternal edge	Adhesions between the heart and sternal halves were lysed. Prevent traction on the right ventricle and rupture. The lower one between the sternal halves The upper one at the subcutaneous level	N/A						
Myllykangas 2022	No	Subcutaneous	Preoperative NPWT in subcutaneous, pectoralis major muscle flap reconstruction. Postoperative iNPWT	N/A						
Akbayrak H 2023	Yes	Substernum	Aggressive sternal and tissue debridement was done. The polyurethane sponge was fitted into the wound substernally	5	5	24	15	1	0	2
Saltarocchi S 2023	No	Between the sternal edge	Polyurethane foam was sized and inserted between the 2 sternal edges. Gray piece of foam was placed over the sternal stumps	N/A						

iNPWT, incisional negative pressure wound therapy; NPWT, negative pressure wound therapy.

### Subgroup analysis

From previous literature, the NPWT was applied for the treatment of DSWI, either as a definite treatment or as a bridge to reconstruction^7,8^. Therefore, we separated our included studies based on the indication of NPWT into three subgroups: NPWT as definite treatment, NPWT as a bridge to reconstruction, and nonmentioned in the study. Two reviewers independently read thoroughly of the articles and separate the articles into subgroup based on the definition described below:

1. NPWT as definite treatment: This category includes studies where NPWT is used as the sole treatment method for DSWI. In these cases, delayed primary wound closure or sternal rewiring following NPWT was arranged. NPWT eliminates the need for further surgical reconstruction.
2. NPWT as bridge to reconstruction: In this category, NPWT is applied to prepare the wound for subsequent surgical procedures. It is not the final treatment but rather a step towards a more comprehensive surgical solution, for instance, staged local flap reconstruction (Pectoralis major muscle flap, omental flap, etc.).
3. Nonmentioned: This category encompasses studies that do not provide enough details about the use of NPWT, whether as a definitive treatment or as a bridge to reconstruction. It also includes studies that apply NPWT in both contexts but do not distinctly categorize them.

Disagreement between the two reviewers were discussed with a third reviewer until a consensus was reached.

### Risk of bias assessment

Study quality was independently assessed by two reviewers utilizing the ROBINS-I tool for nonrandomized studies^18^.

### Statistical analysis

Heterogeneity was assessed using the *I* ^2^ statistic, with values over 50% indicating significant variation with a sensitivity test was conducted. Mortality and reinfection rates were analyzed using risk ratios (RRs), and hospital and ICU stay lengths using mean differences (MDs), each with 95% CIs. Data transformation methods^19,20^ were employed to derive means and SD from median, range, or interquartile ranges (IQR). A random-effects model was utilized to accommodate the retrospective study designs. Analyses were conducted using Review Manager 5.4, with statistical significance set at *P*-value <0.05. For funnel plot interpretation, we conducted Egger’s test with the R programming language^21^.

## Result

A total of 303 relevant studies were initially identified thorough the search. After the title and abstract were reviewed, there were 79 studies remained and the full-text of these studies were evaluated. Finally, 36 studies fulfilled our inclusion criteria and were included in this study^22–58^. Of these studies, all of them were retrospective cohort studies. The characteristic of the eligible studies including main treatment outcome, complication, and indication for NPWT were listed in Table 1. A total of 1981 patients who treated with NPWT, and 1700 patients who received conventional treatment were included in the study. The pool results were analyzed first, and then we divided the treatment outcome into subgroups based on their indication of NPWT, whether as definite treatment, as a bridge to reconstruction or as nonmentioned in the study.

### Primary outcome

#### Mortality rate

**Figure 3:**
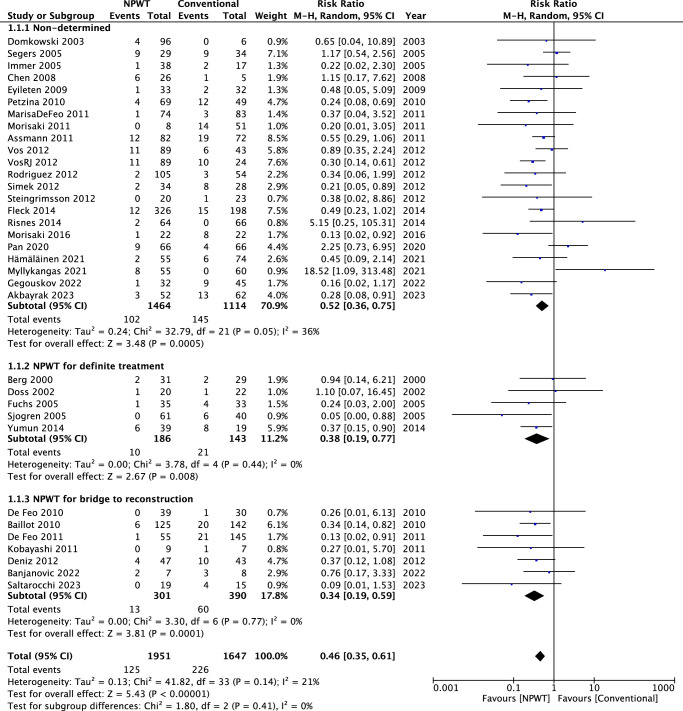
A figure of meta-analysis of mortality rate in patient treated with NPWT and conventional treatment.

In the pooled analysis (34 retrospective studies), there was a significant difference between the NPWT and conventional treatment groups in overall mortality rate (risk ratio 0.46, 95% CI: 0.35–0.61; *P*<0.000001). Low heterogeneity was found among the 34 studies (*I* ^2^=21%; *P*=0.14) (Fig. 3).

In the subgroup analysis of the nondetermined group, no significant heterogeneity was found among the 22 studies (*I* ^2^=36%; *P*=0.05), and there was a significant difference between the NPWT and the conventional treatment group regarding to mortality rate (RR=0.52, 95% CI=0.36–0.75; *P*=0.0005). In the subgroup analysis of NPWT for definite treatment, no significant heterogeneity was found among the five studies (*I* ^2^=0%; *P*=0.44), and there was a significant difference between the NPWT and the conventional treatment group (RR=0.38, 95% CI=0.19–0.77; *P*=0.008). In the subgroup analysis of NPWT for bridge to reconstruction, no significant heterogeneity was found among the seven studies (*I* ^2^=0%; *P*=0.77), and there was significant difference between the NPWT and the conventional treatment group (RR=0.38, 95% CI=0.19–0.59; *P*=0.0001).

#### Reinfection rate

**Figure 4:**
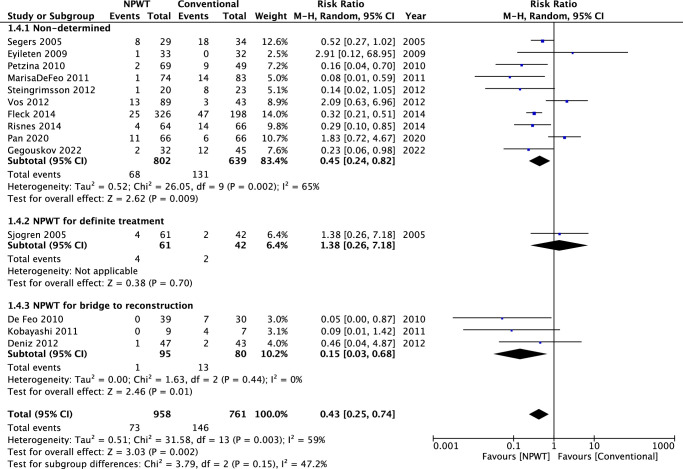
A figure of meta-analysis of reinfection rate in patient treated with NPWT and conventional treatment.

In the pooled analysis (14 retrospective studies), there was significant difference between the NPWT and conventional treatment groups in reinfection rate (RR=0.43, 95% CI: 0.25–0.74; *P*=0.002). Significant heterogeneity was found among the 13 studies (*I* ^2^=59%; *P*=0.003) (Fig. 4).

In the subgroup analysis of the nondetermined group, significant heterogeneity was found among the ten studies (*I* ^2^=65%; *P*=0.002), and there was a significant difference between the NPWT and the conventional treatment group (RR=0.45, 95% CI = 0.24–0.82; *P*=0.009). The subgroup analysis of NPWT for definite treatment is not applicable due to only one study in this subgroup. In the subgroup analysis of NPWT for bridge to reconstruction, no significant heterogeneity was found among the three studies (*I* ^2^=0%; *P*=0.44), and there was a significant difference between the NPWT and the conventional treatment group (RR=0.15, 95% CI=0.03–0.68; *P*=0.01).

### Secondary outcome

#### Length of hospital stay

**Figure 5:**
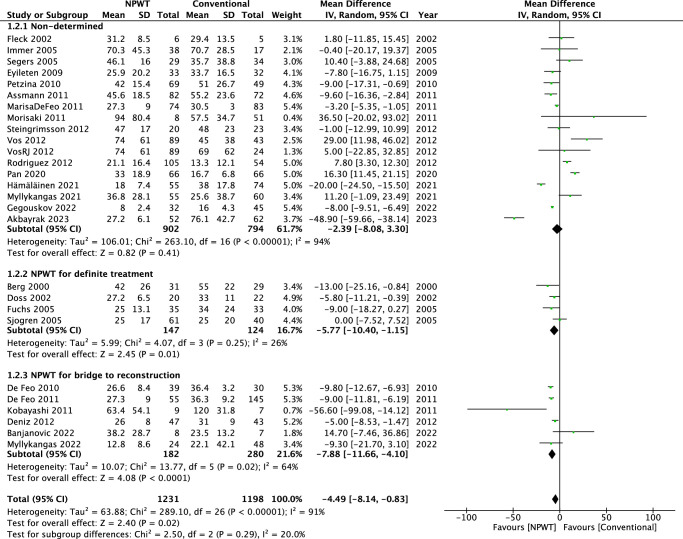
A figure of meta-analysis of length of hospital stay in patient treated with NPWT and conventional treatment.

In the pooled analysis (27 retrospective studies), there was a significant difference between the NPWT and conventional treatment groups in length of hospital stay (MD: −4.49, 95% CI: −8.14 to −0.83; *P*=0.02). Significant heterogeneity was found among the 27 studies (*I* ^2^=91%; *P*<0.00001) (Fig. 5).

In the subgroup analysis of the nondetermined group, significant heterogeneity was found among the 17 studies (*I* ^2^=94%; *P*<0.00001), and there was no significant difference between the NPWT and the conventional treatment group (MD=−2.39, 95% CI=−8.08 to 3.30; *P*=0.41). In the subgroup analysis of NPWT for definite treatment, no significant heterogeneity was found among the four studies (*I* ^2^=26%; *P*=0.25), and there was significant difference between the NPWT and the conventional treatment group (MD=−5.77, 95% CI = −10.40 to −1.15; *P*=0.01). In the subgroup analysis of NPWT for bridge to reconstruction, significant heterogeneity was found among the six studies (*I* ^2^=64%; *P*=0.02), and there was significant difference between the NPWT and the conventional treatment group (MD=−7.88, 95% CI = −11.66 to −4.10; *P*<0.0001).

#### Length of ICU stay

**Figure 6:**
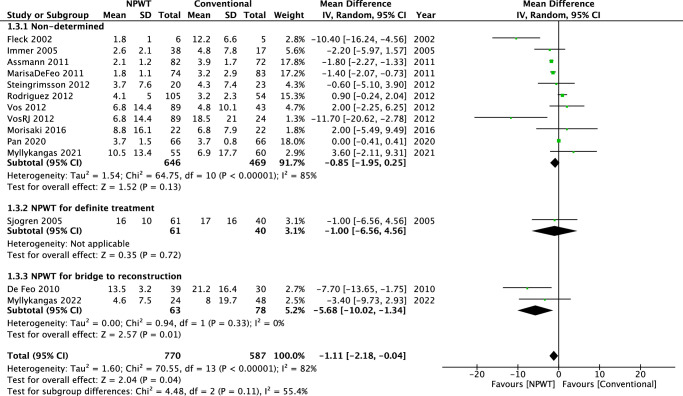
A figure of meta-analysis of length of ICU stay in patient treated with NPWT and conventional treatment.

In the pooled analysis (14 retrospective studies), there was a significant difference between the NPWT and conventional treatment groups in length of ICU stay (mean difference: −1.11, 95% CI: −2.18 to −0.04; *P*=0.04). Significant heterogeneity was found among the 14 studies (*I* ^2^=82%; *P*<0.00001) (Fig. 6).

In the subgroup analysis of the nondetermined group, significant heterogeneity was found among the 11 studies (*I* ^2^=85%; *P*<0.00001), and there was no significant difference between the NPWT and the conventional treatment group (MD=−0.85, 95% CI = −1.95 to 0.25; *P*=0.13). The subgroup analysis of NPWT for definite treatment is not applicable due to only one study in this subgroup. In the subgroup analysis of NPWT for bridge to reconstruction, no significant heterogeneity was found among the two studies (*I* ^2^=0%; *P*=0.33), and there was a significant difference between the NPWT and the conventional treatment group (MD=−5.68, 95% CI=−10.02 to −1.34; *P*=0.01).

#### Sensitivity test

Due to the high level of heterogeneity reported from the result of reinfection rate, hospital stay, and ICU stay, an additional sensitivity test was performed by excluding individual studies from the meta-analysis one by one to assess the robustness of the results. The pooled effect size and heterogeneity remained consistent in mortality rate (RR ranging from 0.43 to 0.48), reinfection rate (RR ranging from 0.37 to 0.47), hospital stay (MD ranging from −5.78 to −2.94), and ICU stay (MD ranging from −1.11 to −0.82), indicating that no single study had a disproportionate influence on the overall findings.

#### Complication

The most frequently reported complications among patients treated with NPWT were right ventricular rupture, major bleeding events, and infections. Right ventricular rupture occurred in seven reported cases across four studies^40,43,52,54^. The likely mechanism for this severe complication is the application of sternal shear forces to the right ventricle. Other complications, though less commonly reported, included major bleeding events^41,53,56,57^, partial flap loss^57^, skin necrosis, wound dehiscence, seroma formation, and atrial fibrillation. Mechanical issues related to NPWT, such as air leakage or retention of foreign material like sponges, were not documented in the included studies. Due to the limited data provided by these individual studies, a meta-analysis of these complications was not feasible.

#### NPWT application information

Table 5 serves to highlight the variability in NPWT settings among studies. Among these studies, the mode setting is generally continuous mode, with pressure set between −75 and −125 mmHg. There are three studies^43,44,50^ acquired intermittent pressure for treatment. The description of wound healing among studies was summarized in Supplementary Digital Supplement 6, http://links.lww.com/JS9/D535.

**Table 5:** Negative pressure wound therapy application information.

First author/Year	Mode	Pressure (mmHg)	Mean duration of application (days, mean/SD)	Brand of NPWT	Frequency of changes (days)
Berg 2000	Continuous	−300 to −600	N/A	N/A	N/A
Doss 2002	Continuous	−125	N/A	KCI Medical, San Antonio, TX	2–3
Fleck TM 2002	Continuous	−75 to −125	9.3/3.18	N/A	2–3
Domkowski 2003	Continuous	−125	N/A	N/A	N/A
Fuchs 2005	Continuous	−125 to −150, −75 if pain	N/A	N/A	3–7
Immer 2005	N/A	−75 to −150	N/A	KCI Medical, San Antonio, TX	2–3
Segers 2005	Continuous	−125	N/A	N/A	4–5
Sjogren 2005	N/A	N/A	12/9	N/A	N/A
Chen Y 2008	Intermittent	−125	18.23/16.15	KCI Medical, San Antonio, TX	2–3
Eyileten 2009	Intermittent	−75 to −100	N/A	N/A	2–3
Baillot R 2010	Continuous	−75 to −125	15/7.7	KCI Medical, San Antonio, TX	2–3
De Feo 2010	Continuous	−125	N/A	KCI Medical, San Antonio, TX	2–3
Petzina 2010	Continuous	−125	5.5/1.6	N/A	2–4
Assmann 2011	N/A	N/A	N/A	KCI, Wies-badan, Germany	N/A
Marisa De Feo 2011	Continuous	−125	12.6/5.5	KCI Medical, San Antonio, TX	2–3
De Feo 2011	Continuous	−125	15.2/5.2	KCI Medical, San Antonio, TX	2–3
Kobayashi 2011	Continuous	−100 to −120	22.6/11.7	3M Healthcare; St. Paul, MN	2–7
Morisaki 2011	N/A	N/A	N/A	Ethicon, Somerville, NJ, USA	2–3
Deniz 2012	Intermittent	−75 to −125	N/A	KCI Medical, San Antonio, TX	2–3
Rodriguez CB 2012	N/A	−50 to −100	N/A	N/A	4–5
Simek 2012	N/A	N/A	N/A	N/A	N/A
Steingrimsson 2012	N/A	−125	N/A	KCI Medical, San Antonio, TX	2–4
Vos RJ 2012	Continuous	−75 to −125	N/A	KCI Medical, San Antonio, TX	3–4
Vos 2012	Continuous	−75 to −125	N/A	N/A	3–4
Fleck T 2014	Continuous	−125	11/8	KCI Medical, San Antonio, TX	2–3
Risnes I 2014	Continuous	−125	25.8/20.5	KCI, Copenhagen, Denmark	N/A
Yumun G 2014	N/A	N/A	16.8/8	N/A	2
Morisaki 2016	Continuous	−75 to −125	N/A	KCI Medical, San Antonio, TX	2–4
Pan T 2020	N/A	−75 to −125	N/A	KCI Medical, San Antonio, TX	2–3
Hämäläinen E 2021	N/A	N/A	N/A	N/A	N/A
Myllykangas HM 2021	N/A	N/A	N/A	N/A	N/A
Banjanovic B 2022	N/A	N/A	N/A	N/A	N/A
Gegouskov V 2022	Continuous or intermittent	−50 to −125	7	N/A	2–4
Myllykangas HM 2022	N/A	N/A	N/A	KCI Medical, San Antonio, TX	3
Akbayrak H 2023	Continuous	−75 to −150	N/A	N/A	N/A
Saltarocchi S 2023	N/A	−125	N/A	TheraFlo (3M+KCI)	5

#### Funnel plot

**Figure 7:**
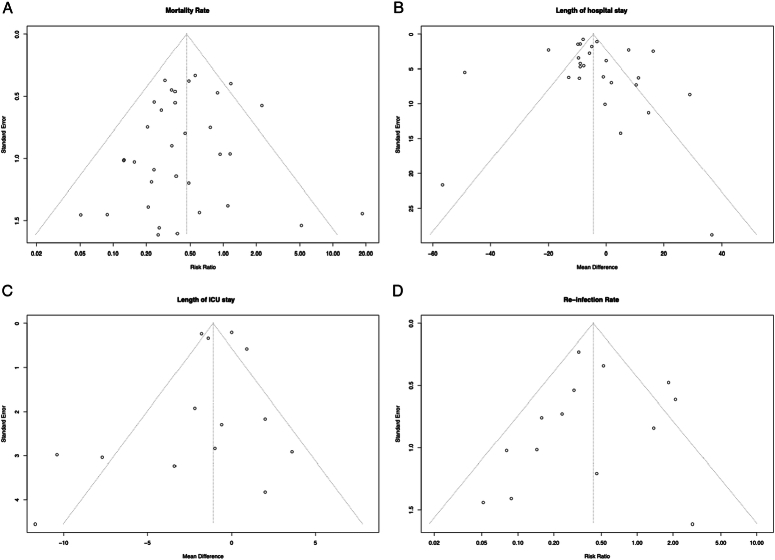
A figure of funnel plot of each treatment outcome (a) mortality, (b) length of hospital stay, (c) length of ICU stay, and (d) reinfection rate.

Egger’s test^59,60^ was conducted to assess publication bias. The test yielded a *P*-value of 0.49, 0.79, 0.48, 0.5 for result of mortality, reinfection, length of hospital stay, and length of ICU stay, respectively, indicating no significant evidence of bias (*P*>0.05). A visual inspection of the funnel plot confirmed the absence of asymmetry, further supporting this conclusion (Fig. 7).

#### Risk of bias assessment

**Figure 8:**
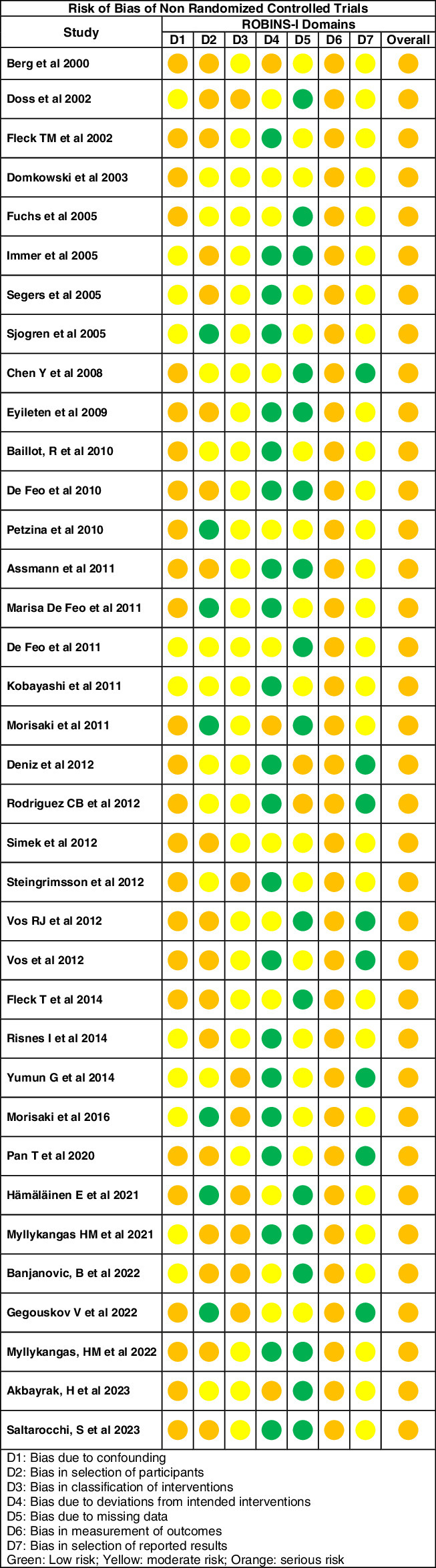
A figure of summary of the Risk of Bias of Nonrandomized Studies of Intervention (ROBINS-I) assessment.

In evaluating the effectiveness and safety of NPWT for DSWI, the ROBINS-I framework revealed a nuanced spectrum of bias across the included studies. The majority exhibited low to moderate risk in domains such as bias due to deviations from intended interventions, bias due to missing data, and bias in the selection of reported results, indicating a generally reliable evidence base. However, some studies showed a serious risk in domains like bias due to confounding and bias in the measurement of outcomes, suggesting potential inconsistencies in NPWT application or adherence. Detailed results are presented in the Supplemental Digital Content 5, http://links.lww.com/JS9/D534 and summarized for each study in Figure 8. This variation underscores the importance of standardized protocols and rigorous study designs in future research. Overall, the ROBINS-I assessment highlights the potential of NPWT but calls for careful consideration of identified biases to enhance the validity of conclusions regarding its efficacy and safety.

## Discussion

Our study encompasses a total of 36 retrospective studies, making it the largest systematic review on this topic to date with an aggregate participant count of ~3600 cases. These findings indicate a significant reduction in mortality and reinfection rates, as well as decreased lengths of hospital and ICU stays in patients treated with NPWT compared to those receiving conventional treatments. Previous meta-analyses by Falagas *et al*.^12^ in 2013 and Damiani *et al*.^11^ in 2011 have established the efficacy of NPWT in reducing mortality and shortening hospital stay durations. However, the NPWT device advanced in the past decade such as the NPWT instillation and dwell^61^, which was not discussed in previous studies. Besides, their studies did not explore ICU stay lengths and reinfection rates due to limited data. To our knowledge, this is the first study providing a comprehensive analysis of NPWT, including these additional outcome measures, compared to conventional treatment for DSWI. In addition, subgroup analyses were conducted to offer a more in-depth review of the clinical use of NPWT for DSWI across different clinical indications.

In our analysis, we observed a broad spectrum of conventional treatments across the studies, including debridement, closed irrigation, open packing, and reconstruction using omental, pectoral flaps, or other flaps. Given this variability, high clinical heterogeneity was anticipated. To address this, we employed a random-effects model and conducted subgroup analyses based on the specific application of NPWT, whether as a bridge therapy or as a definitive treatment. We categorized eight studies as ‘bridge to reconstruction'^45,47–50,54,55,58^ and 5 as ‘definitive treatment'^37,38,40,42,53^ after a thorough review.

In subgroup analysis, the efficacy and safety of NPWT as a bridge therapy were substantiated. This modality was associated with improved outcomes, including reduced mortality (RR=0.38, *P*=0.0001), lower reinfection rates (RR=0.15, *P*=0.01), and decreased lengths of the hospital (MD=−7.88 days, *P*<0.0001) and ICU stays (MD=−5.68 days, *P*=0.01).

Among the eight included studies that applied NPWT as a bridge therapy, Baillot *et al*.^45^ reported on the outcomes in patients with DSWI managed with NPWT as a precursor to sternal osteosynthesis using horizontal titanium plates and pectoralis major muscle flap (PMMF) coverage. De Feo *et al*.^47,48^, as well as Myllykangas *et al*.^57^, utilized NPWT to facilitate subsequent sternal wound reconstruction with PMMF. Kobayashi *et al*.^49^ achieved sternal wound closure with a secondary omental flap and PMMF, while Deniz *et al*.^50^ integrated NPWT into a broader therapeutic framework as a transition to flap reconstruction. Banjanovic *et al*.^55^ and Saltarocchi *et al*.^58^ deployed NPWT as an interim solution prior to reconstruction with PMMF and sternum refixation, including the application of Nitinol clips.

Furthermore, NPWT as a definitive treatment also demonstrated significant benefits, yielding lower mortality (RR=0.38, *P*=0.008) and reduced hospitalization duration (MD=−5.77, *P*=0.01) compared to conventional therapy. Berg *et al*.^37^ contrasted continuous irrigation and vacuum drainage as initial treatments for postcardiac surgery mediastinitis. Doss *et al*.^38^ continued NPWT until adequate granulation tissue developed, followed by primary wound closure. Fuchs *et al*.^40^ and Sjogren *et al*.^42^ employed NPWT as the sole therapy, foregoing muscle flaps or omentoplasty, and proceeding to sternal rewiring. Yumun G *et al*.^53^ used NPWT in conjunction with sternal rewiring using stainless steel sutures and performed the Robicsek procedure as required.

The favorable outcomes observed in these subgroups may be attributed to the inherent benefits of NPWT. Initial studies^62–65^ have demonstrated that NPWT facilitates exudate removal, augments blood flow in surrounding tissues, and contributes to bacterial count reduction, leading to enhanced healing and granulation. Consequently, this may result in lower reinfection rates and mortality. Moreover, NPWT serves to approximate wound edges, fill tissue gaps, and stabilize the sternum, thereby minimizing the need for frequent dressing changes and promoting earlier mobilization and extubation. This enables patients to transition to the general ward sooner, reducing intensive care requirements. Two comprehensive reviews^66,67^ have endorsed NPWT’s superiority over conventional treatment, suggesting its role as either definitive care or a bridge to musculocutaneous reconstruction in managing poststernotomy DSWI. For clinical implication, our results further support the application of NPWT in both clinical scenarios and analyze the additional outcome including reinfection rate and length of ICU stay, which were not analyzed in previous literatures.

Our findings align with prior research^8,68^ advocating NPWT as a bridge therapy for DSWI; however, we have not addressed the optimal timing for wound reconstruction. Levy *et al*.^69,70^ advocate for early sternal wound reconstruction within 30 days, demonstrating improved postoperative infection and wound complication rates compared to delayed reconstruction (beyond 30 days). Similarly, Qiu X *et al*. conducted a meta-analysis^71^ concluded that immediate flap reconstruction correlates with lower in-hospital mortality and a shorter hospital stay than NPWT alone. Therefore, reconstruction should proceed without delay once the wound is clean and conducive to surgery. Should staged reconstruction be necessary due to factors such as inadequate debridement, persistent infection, or hemodynamic instability, NPWT is recommended as a bridge therapy to reduce mortality, reinfection rates, and length of hospitalization^72^. Right ventricular (RV) rupture is an infrequent yet potentially life-threatening complication following poststernotomy mediastinitis. Abu-Omar *et al*.^73^ reported two instances of RV rupture related to the use of negative pressure wound therapy (NPWT). They hypothesized that RV wall rupture could result from the excessive stretching of the RV, adherent to the chest wall and sternum, during abrupt increases in intrathoracic pressure (cough or vomiting). To prevent this grave complication, several measures are suggested, including thorough mobilization of the RV during initial debridement, applying paraffin gauze over the RV surface, application of a HeartShield device^74^, and consistently using polyurethane foam to mitigate shearing forces during sudden chest movements. Johan Sjogren *et al*.^4^ documented their clinical experiences with NPWT-associated RV rupture and major bleeding events. Their findings indicated that while RV rupture-induced bleeding occurred in 2.3% of patients, the overall 30-day mortality rate was 1.1%. Their analysis concluded that the advantages of NPWT generally surpass the risk of RV rupture. Echoing Abu-Omar *et al*.^73^, they advocated for the protective use of paraffin gauze between the RV and the polyurethane foam. To circumvent severe complications, it is imperative to adhere strictly to the manufacturer’s guidelines and to exercise caution when contraindications to NPWT are present, including ongoing infection, untreated osteomyelitis, malignancy in the wound, nonenteric fistulas, and bleeding disorders.

### Limitation

Firstly, it is important to acknowledge that our review exclusively encompasses retrospective chart reviews, which inherently carry risks of nonrandomization of patients, interpretive errors, incomplete data collection, and potential investigator bias. Given the heterogeneity within the control groups, asserting the unequivocal superiority of NPWT over all conventional treatment modalities remains challenging^66^. In addition, the potential reasons for the high heterogeneity of results include the variety in patient population, intervention, and the different definitions of reinfection across studies. Bain *et al*.^75^ critique the conclusions of previous meta-analyses and caution against a default ‘Try VAC first’ strategy in managing DSWI. To address these concerns, our meta-analysis conducted a nuanced subgroup analysis, stratifying studies based on the specific indications for NPWT. The results of this detailed examination add a robust layer of evidence, reinforcing the utility of NPWT in the treatment of DSWI, which we believe represents the most compelling evidence available to date.

Second, this study conducted a subgroup analysis on the included articles, dividing them into three categories: nonmentioned, bridge to reconstruction, and definite treatment. However, the nonmentioned group constituted the largest proportion, as many articles did not provide sufficient information. As a result, we were unable to analyze the results from the nonmentioned group.

This review also considers the diversity of NPWT systems, acknowledging variations among manufacturers, treatment durations, specific pressure settings, and the new generation of NPWT, such as those combining irrigation systems. The deployment details of NPWT across studies are consolidated in Table 5. Most studies use 3M’s V.A.C. system for treating deep sternal infections. We believe this is primarily due to 3M’s global leadership in the negative pressure wound therapy market^76^, leading many institutions to adopt this device for research. At the same time, we should be cautious to ensure that each study honestly discloses any conflicts of interest to avoid potential bias in the research results. An earlier animal study^63^ suggested that negative pressures of −75 or −100 mmHg can promote peri-sternal soft tissue blood flow, beneficial in DSWI treatment. However, the influence of these variable treatment parameters on patient outcomes remains uncertain, underlining the need for further randomized controlled trials to standardize these variables and provide a higher level of evidence for this conclusion.

## Conclusion

The state-of-art of this meta-analysis suggest that negative pressure wound therapy can reduce mortality and reinfection rate, shorten hospital, and ICU stay in patients with deep sternal wound infection after cardiovascular surgery. Due to the retrospective design of the included studies, further randomized controlled trials are needed to provide a higher level of evidence for this conclusion.

## Ethical approval

Not applicable.

## Consent

Not applicable.

## Source of funding

This research did not receive any funding source.

## Author contribution

C.-H.L., Y.-T.L., and C.-H.L.: study concept or design; Y.-T.L. and S.-H.L.: data collection; Y.-T.L., S.-H.L., and C.P.: data analysis and interpretation; Y.-T.L. and S.-H.L.: original draft writing; C.-H.L. and R.-W.H.: critical review and editing; C.-H.L., S.-H.C., and C.-C.H.: supervision; Y.-T.L.: project administration.

## Conflicts of interest disclosure

The authors declare no conflicts of interest.

## Research registration unique identifying number (UIN)

1. Name of the registry: PROSPERO.
2. Unique Identifying number or registration ID: CRD42024470569.
3. Hyperlink to your specific registration (must be publicly accessible and will be checked):https://www.crd.york.ac.uk/prospero/display_record.php?ID=CRD42024470569

1. Name of the registry: Research Registry.
2. Unique Identifying number or registration ID: reviewregistry1894.
3. Hyperlink to your specific registration (must be publicly accessible and will be checked): https://researchregistry.knack.com/research-registry#registryofsystematicreviewsmetaanalyses/registryofsystematicreviewsmeta-analysesdetails/67029700b6c74802e2881ecb/.

## Guarantor

Che-Hsiung Lee, M.D.

## Data availability statement

The data that support the findings of this study are openly available in published articles. The included articles and reference are listed in Table 1.

## Provenance and peer review

Not commissioned, externally peer-reviewed.

## Supplementary Material

SUPPLEMENTARY MATERIAL
