# Supplementary material for: Effectiveness and safety of negative pressure wound therapy in patients with deep sternal wound infection: a systematic review and meta-analysis
Source: Int J Surg. 2024 Nov 14;110(12):8107–25. doi: 10.1097/JS9.0000000000002138 (PMC11634157; doi:10.1097/JS9.0000000000002138)
Supplement: SUPPLEMENTARY MATERIAL [file js9-110-8107-s003.pdf]

Supplemental Digital Content 3 _PubMed Search History

Search number	Query	Search Details	Results
**20**	#7 and #15 and #19	("negative pressure"[Title/Abstract] OR "VAC"[All Fields] OR ("negative pressure wound therapy"[MeSH Terms] OR ("negative pressure"[All Fields] AND "wound"[All Fields] AND "therapy"[All Fields]) OR "negative pressure wound therapy"[All Fields] OR ("vacuum"[All Fields] AND "assisted"[All Fields] AND "closure"[All Fields]) OR "vacuum assisted closure"[All Fields]) OR ("negative pressure wound therapy"[MeSH Terms] OR "negative pressure"[All Fields] OR "vacuum assisted closure"[All Fields]) OR "vacuum assisted closure"[Title/Abstract]) AND ((("sternum"[MeSH Terms] OR "sternum"[All Fields] OR "sternal"[All Fields]) AND ("wound infection"[MeSH Terms] OR ("wound"[All Fields] AND "infection"[All Fields]) OR "wound infection"[All Fields])) OR "sternal wound infection"[Title/Abstract] OR "dswi"[Title/Abstract] OR "deep sternal wound infection"[Title/Abstract] OR ("mediastin"[All Fields] OR "mediastinally"[All Fields] OR "mediastine"[All Fields] OR "mediastinic"[All Fields] OR "mediastinitis"[MeSH Terms] OR "mediastinitis"[All Fields] OR "mediastinum"[MeSH Terms] OR "mediastinum"[All Fields] OR "mediastinal"[All Fields]) OR ("osteomyelities"[All Fields] OR "osteomyelitis"[MeSH Terms] OR "osteomyelitis"[All Fields] OR "osteomyelitides"[All Fields])) AND ("mortality"[MeSH Terms] OR "mortality"[All Fields] OR ("mortality"[All Fields] AND "rate"[All Fields]) OR "mortality rate"[All Fields] OR (("reinfection"[MeSH Terms] OR "reinfection"[All Fields] OR ("re"[All Fields] AND "infection"[All Fields]) OR "re infection"[All Fields]) AND ("j rehabil assist technol eng"[Journal] OR "rate"[All Fields])) OR ("length of stay"[MeSH Terms] OR ("length"[All Fields] AND "stay"[All Fields]) OR "length of stay"[All Fields] OR ("length"[All Fields] AND "hospital"[All Fields] AND "stay"[All Fields]) OR "length of hospital stay"[All Fields]))	228
**19**	#16 or #17 or #18	"mortality"[MeSH Terms] OR "mortality"[All Fields] OR ("mortality"[All Fields] AND "rate"[All Fields]) OR "mortality rate"[All Fields] OR (("reinfection"[MeSH Terms] OR "reinfection"[All Fields] OR ("re"[All Fields] AND "infection"[All Fields]) OR "re infection"[All Fields]) AND ("j rehabil assist technol eng"[Journal] OR "rate"[All Fields])) OR ("length of stay"[MeSH Terms] OR ("length"[All Fields] AND "stay"[All Fields]) OR "length of stay"[All Fields] OR ("length"[All Fields] AND "hospital"[All Fields] AND "stay"[All Fields]) OR "length of hospital stay"[All Fields])	1,637,326
**18**	length of hospital stay	"length of stay"[MeSH Terms] OR ("length"[All Fields] AND "stay"[All Fields]) OR "length of stay"[All Fields] OR ("length"[All Fields] AND "hospital"[All Fields] AND "stay"[All Fields]) OR "length of hospital stay"[All Fields]	172,710
**17**	re-infection rate	("reinfection"[MeSH Terms] OR "reinfection"[All Fields] OR ("re"[All Fields] AND "infection"[All Fields]) OR "re infection"[All Fields]) AND ("j rehabil assist technol eng"[Journal] OR "rate"[All Fields])	6,166
**16**	mortality rate	"mortality"[MeSH Terms] OR "mortality"[All Fields] OR ("mortality"[All Fields] AND "rate"[All Fields]) OR "mortality rate"[All Fields]	1,522,986
**15**	#8 or #9 or #10 or #12 or #13 or #14	(("sternum"[MeSH Terms] OR "sternum"[All Fields] OR "sternal"[All Fields]) AND ("wound infection"[MeSH Terms] OR ("wound"[All Fields] AND "infection"[All Fields]) OR "wound infection"[All Fields])) OR "sternal wound infection"[Title/Abstract] OR "dswi"[Title/Abstract] OR "deep sternal wound infection"[Title/Abstract] OR ("mediastin"[All Fields] OR "mediastinally"[All Fields] OR "mediastine"[All Fields] OR "mediastinic"[All Fields] OR "mediastinitis"[MeSH Terms] OR "mediastinitis"[All Fields] OR "mediastinum"[MeSH Terms] OR "mediastinum"[All Fields] OR "mediastinal"[All Fields]) OR ("osteomyelities"[All Fields] OR "osteomyelitis"[MeSH Terms] OR "osteomyelitis"[All Fields] OR "osteomyelitides"[All Fields])	105,765
**14**	osteomyelitis	"osteomyelities"[All Fields] OR "osteomyelitis"[MeSH Terms] OR "osteomyelitis"[All Fields] OR "osteomyelitides"[All Fields]	34,780
**13**	mediastinitis	"mediastin"[All Fields] OR "mediastinally"[All Fields] OR "mediastine"[All Fields] OR "mediastinic"[All Fields] OR "mediastinitis"[MeSH Terms] OR "mediastinitis"[All Fields] OR "mediastinum"[MeSH Terms] OR "mediastinum"[All Fields] OR "mediastinal"[All Fields]	69,591
**12**	Deep sternal wound infection[Title/Abstract]	"deep sternal wound infection"[Title/Abstract]	682
**11**	Deep sternal wound infection	"Deep"[All Fields] AND ("sternum"[MeSH Terms] OR "sternum"[All Fields] OR "sternal"[All Fields]) AND ("wound infection"[MeSH Terms] OR ("wound"[All Fields] AND "infection"[All Fields]) OR "wound infection"[All Fields])	1,020
**10**	dswi[Title/Abstract]	"dswi"[Title/Abstract]	248
**9**	sternal wound infection[Title/Abstract]	"sternal wound infection"[Title/Abstract]	1,209
**8**	sternal wound infection	("sternum"[MeSH Terms] OR "sternum"[All Fields] OR "sternal"[All Fields]) AND ("wound infection"[MeSH Terms] OR ("wound"[All Fields] AND "infection"[All Fields]) OR "wound infection"[All Fields])	2,611
**7**	#2 or #3 or #4 or #5 or #6	"negative pressure"[Title/Abstract] OR "VAC"[All Fields] OR ("negative pressure wound therapy"[MeSH Terms] OR ("negative pressure"[All Fields] AND "wound"[All Fields] AND "therapy"[All Fields]) OR "negative pressure wound therapy"[All Fields] OR ("vacuum"[All Fields] AND "assisted"[All Fields] AND "closure"[All Fields]) OR "vacuum assisted closure"[All Fields]) OR ("negative pressure wound therapy"[MeSH Terms] OR "negative pressure"[All Fields] OR "vacuum assisted closure"[All Fields]) OR "vacuum assisted closure"[Title/Abstract]	22,006
**6**	vacuum assisted closure[Title/Abstract]	"vacuum assisted closure"[Title/Abstract]	1,633
**5**	negative pressure wound therapy[MeSH Terms] OR "negative pressure"[All Fields] OR "vacuum assisted closure"[All Fields]	"negative pressure wound therapy"[MeSH Terms] OR "negative pressure"[All Fields] OR "vacuum assisted closure"[All Fields]	14,017
**4**	vacuum assisted closure	"negative pressure wound therapy"[MeSH Terms] OR ("negative pressure"[All Fields] AND "wound"[All Fields] AND "therapy"[All Fields]) OR "negative pressure wound therapy"[All Fields] OR ("vacuum"[All Fields] AND "assisted"[All Fields] AND "closure"[All Fields]) OR "vacuum assisted closure"[All Fields]	6,351
**3**	VAC	"VAC"[All Fields]	9,091
**2**	negative pressure[Title/Abstract]	"negative pressure"[Title/Abstract]	11,447
**1**	negative pressure	("negative"[All Fields] OR "negatively"[All Fields] OR "negatives"[All Fields] OR "negativities"[All Fields] OR "negativity"[All Fields]) AND ("pressure"[MeSH Terms] OR "pressure"[All Fields] OR "pressures"[All Fields] OR "pressure s"[All Fields] OR "pressurisation"[All Fields] OR "pressurised"[All Fields] OR "pressuriser"[All Fields] OR "pressurization"[All Fields] OR "pressurizations"[All Fields] OR "pressurize"[All Fields] OR "pressurized"[All Fields] OR "pressurizer"[All Fields] OR "pressurizes"[All Fields] OR "pressurizing"[All Fields])	72,266
