# Supplementary material for: Effectiveness and safety of negative pressure wound therapy in patients with deep sternal wound infection: a systematic review and meta-analysis
Source: Int J Surg. 2024 Nov 14;110(12):8107–25. doi: 10.1097/JS9.0000000000002138 (PMC11634157; doi:10.1097/JS9.0000000000002138)
Supplement: SUPPLEMENTARY MATERIAL [file js9-110-8107-s004.pdf]

Supplemental Digital Content 4_Embase Search History

No.	Query	Results
#4	('negative pressure'/exp OR 'vacuum assisted closure'/exp OR 'negative pressure dressing' OR 'negative pressure dressings' OR 'negative pressure therapy' OR 'negative pressure wound therapy' OR 'negative-pressure wound therapy' OR 'topical negative pressure' OR 'vacuum assisted closure' OR 'vacuum assisted wound closure' OR 'vacuum therapy' OR 'negative pressure wound therapy':ti,ab) AND ('sternal wound infection'/exp OR 'deep sternal wound infection'/exp OR dswi:ti,ab OR 'mediastinitis'/exp OR 'fibrous mediastinitis':ti,ab,kw OR 'mediastinitis':ti,ab,kw OR 'mediastinum infection':ti,ab,kw OR 'osteomyelitis'/exp OR 'osteomyelitis':ti,ab,kw) AND ('mortality rate'/exp OR 'death rate':ti,ab,kw OR 'death rate model':ti,ab,kw OR 'fatal outcome rate':ti,ab,kw OR 'fatality rate':ti,ab,kw OR 'lethal outcome rate':ti,ab,kw OR 'mortality rate':ti,ab,kw OR 'rate, mortality':ti,ab,kw OR 're-infection rate':ti,ab OR 'length of hospital stay':ti,ab)	71
#3	'mortality rate'/exp OR 'death rate':ti,ab,kw OR 'death rate model':ti,ab,kw OR 'fatal outcome rate':ti,ab,kw OR 'fatality rate':ti,ab,kw OR 'lethal outcome rate':ti,ab,kw OR 'mortality rate':ti,ab,kw OR 'rate, mortality':ti,ab,kw OR 're-infection rate':ti,ab OR 'length of hospital stay':ti,ab	318985
#2	'sternal wound infection'/exp OR 'deep sternal wound infection'/exp OR dswi:ti,ab OR 'mediastinitis'/exp OR 'fibrous mediastinitis':ti,ab,kw OR 'mediastinitis':ti,ab,kw OR 'mediastinum infection':ti,ab,kw OR 'osteomyelitis'/exp OR 'osteomyelitis':ti,ab,kw	67568
#1	'negative pressure'/exp OR 'vacuum assisted closure'/exp OR 'negative pressure dressing' OR 'negative pressure dressings' OR 'negative pressure therapy' OR 'negative pressure wound therapy' OR 'negative-pressure wound therapy' OR 'topical negative pressure' OR 'vacuum assisted closure' OR 'vacuum assisted wound closure' OR 'vacuum therapy' OR 'negative pressure wound therapy':ti,ab	11805
