# Supplementary material for: Effectiveness and safety of negative pressure wound therapy in patients with deep sternal wound infection: a systematic review and meta-analysis
Source: Int J Surg. 2024 Nov 14;110(12):8107–25. doi: 10.1097/JS9.0000000000002138 (PMC11634157; doi:10.1097/JS9.0000000000002138)
Supplement: SUPPLEMENTARY MATERIAL [file js9-110-8107-s005.docx]

**Summary of risk of bias in non randomized controlled studies**

| **Title: Berg 2000**  **Type of study:** Retrospective study  **Participants:** mediastinitis following open cardiac surgery  **Surgical intervention:** definite treatment with vacuum assisted closure  **Conservative treatment:** continuous irrigation | | | | | | | | | |
| --- | --- | --- | --- | --- | --- | --- | --- | --- | --- |
| **Outcome** | **Benefit or harm of intervention** | **Domains** | | | | | | | **Overall Risk of Bias** |
|  |  | **Bias due to confounding** | **Bias in selection of participants into the study** | **Bias in classification of interventions** | **Bias due to deviations from intended interventions** | **Bias due to missing data** | **Bias in measurement of outcomes** | **Bias in selection of the reported result** |  |
| **Mortality** | **Benefit** | **Serious^1^** | **Serious^2^** | **Moderate** | **Serious^3^** | **Moderate** | **Serious^4^** | **Moderate** | **Serious** |
| **Explanatory Footnotes**  1. Bias due to confounding because some potential confounders like age, sex, duration of therapy time, time from surgery, and different degree of infection involvement  2. Bias in selection of participants into the study because choices were made by surgeon’s decision instead of historical or pre-interventions  3. Bias due to deviations from intended interventions of large vaccum pressure differences  4. Bias due to measurement of outcomes because results of this study cannot be double blinded for the outcomes accessor | | | | | | | | | |

| **Title: Doss 2002**  **Type of study:** Retrospective study  **Participants:** Post-sternotomy osteomyelitis  **Surgical intervention:** definite treatment with vacuum assisted closure  **Conservative treatment:** re-exploration, debridement, irrigation and open drainage | | | | | | | | | |
| --- | --- | --- | --- | --- | --- | --- | --- | --- | --- |
| **Outcome** | **Benefit or harm of intervention** | **Domains** | | | | | | | **Overall Risk of Bias** |
|  |  | **Bias due to confounding** | **Bias in selection of participants into the study** | **Bias in classification of interventions** | **Bias due to deviations from intended interventions** | **Bias due to missing data** | **Bias in measurement of outcomes** | **Bias in selection of the reported result** |  |
| **Mortality** | **Benefit** | **Moderate** | **Serious^1^** | **Serious^2^** | **Moderate** | **Low^2^** | **Serious^3^** | **Moderate** | **Serious** |
| **Explanatory Footnotes**  1. Bias in selection of participants into the study because choices were made by surgeon’s decision instead of historical or pre-interventions  2. Bias due to classification of interventions because data information extracted from medical records  3. Bias due to measurement of outcomes because results of this study cannot be double blinded as an outcomes accessor | | | | | | | | | |

| **Title: Fleck 2002**  **Type of study:** Retrospective study  **Participants:** deep sternal wound infection following open cardiac surgery  **Surgical intervention:** bridging of NPWT system follow wth muscle flap reconstruction  **Conservative treatment:** primary closure after debridement | | | | | | | | | |
| --- | --- | --- | --- | --- | --- | --- | --- | --- | --- |
| **Outcome** | **Benefit or harm of intervention** | **Domains** | | | | | | | **Overall Risk of Bias** |
|  |  | **Bias due to confounding** | **Bias in selection of participants into the study** | **Bias in classification of interventions** | **Bias due to deviations from intended interventions** | **Bias due to missing data** | **Bias in measurement of outcomes** | **Bias in selection of the reported result** |  |
| **Mortality** | **Benefit** | **Serious^1^** | **Serious^2^** | **Moderate** | **Low** | **Moderate** | **Serious^3^** | **Moderate** | **Serious** |
| **Explanatory Footnotes**  1. Bias due to confounding because some potential confounders like age, sex, duration of therapy time, time from surgery, and different degree of infection involvement  2. Bias in selection of participants into the study because choices were made by surgeon’s decision instead of historical or pre-interventions  3. Bias due to measurement of outcomes because results of this study cannot be double blinded as an outcomes accessor | | | | | | | | | |

| **Title: Domkowski 2003**  **Type of study:** Retrospective study  **Participants:** mediastinitis following open cardiac surgery  **Surgical intervention:** definite and bridging treatment of NPWT  **Conservative treatment:** Multiple dressing change | | | | | | | | | |
| --- | --- | --- | --- | --- | --- | --- | --- | --- | --- |
| **Outcome** | **Benefit or harm of intervention** | **Domains** | | | | | | | **Overall Risk of Bias** |
|  |  | **Bias due to confounding** | **Bias in selection of participants into the study** | **Bias in classification of interventions** | **Bias due to deviations from intended interventions** | **Bias due to missing data** | **Bias in measurement of outcomes** | **Bias in selection of the reported result** |  |
| **Mortality** | **Benefit** | **Serious^1^** | **Moderate** | **Moderate** | **Moderate** | **Moderate** | **Serious^2^** | **Moderate** | **Serious** |
| **Explanatory Footnotes**  1. Bias due to confounding because some potential confounders like age, sex, duration of therapy time, time from surgery, and different degree of infection involvement  2. Bias due to measurement of outcomes because results of this study cannot be double blinded as an outcomes accessor | | | | | | | | | |

| **Title: Fuchs 2005**  **Type of study:** Retrospective study  **Participants:** deep sternal wound infection following open cardiac surgery  **Surgical intervention:** rewiring and closure after NPWT  **Conservative treatment:** irrigation, wound drainage, open packing, delayed closure | | | | | | | | | |
| --- | --- | --- | --- | --- | --- | --- | --- | --- | --- |
| **Outcome** | **Benefit or harm of intervention** | **Domains** | | | | | | | **Overall Risk of Bias** |
|  |  | **Bias due to confounding** | **Bias in selection of participants into the study** | **Bias in classification of interventions** | **Bias due to deviations from intended interventions** | **Bias due to missing data** | **Bias in measurement of outcomes** | **Bias in selection of the reported result** |  |
| **Mortality** | **Benefit** | **Serious^1^** | **Moderate** | **Moderate** | **Moderate** | **Low** | **Serious^2^** | **Moderate** | **Serious** |
| **Explanatory Footnotes**  1. Bias due to confounding because some potential confounders like age, sex, duration of therapy time, time from surgery, and different degree of infection involvement  2. Bias due to measurement of outcomes because results of this study cannot be double blinded as an outcomes accessor | | | | | | | | | |

| **Title: Immer 2005**  **Type of study:** Retrospective study  **Participants:** deep sternal wound infection following open cardiac surgery  **Surgical intervention:** definite and bridging treatment of NPWT  **Conservative treatment:** sternal excision, primary musculocutaneous flap | | | | | | | | | |
| --- | --- | --- | --- | --- | --- | --- | --- | --- | --- |
| **Outcome** | **Benefit or harm of intervention** | **Domains** | | | | | | | **Overall Risk of Bias** |
|  |  | **Bias due to confounding** | **Bias in selection of participants into the study** | **Bias in classification of interventions** | **Bias due to deviations from intended interventions** | **Bias due to missing data** | **Bias in measurement of outcomes** | **Bias in selection of the reported result** |  |
| **Mortality** | **Benefit** | **Moderate** | **Serious^1^** | **Moderate** | **Low** | **Low** | **Serious^2^** | **Moderate** | **Serious** |
| **Explanatory Footnotes**  1. Bias in selection of participants into the study because choices were made by surgeon’s decision instead of historical or pre-interventions  2. Bias due to measurement outcomes because outcomes assessor was not clearly stated | | | | | | | | | |

| **Title: Segers 2005**  **Type of study:** Retrospective study  **Participants:** mediastinitis following open cardiac surgery  **Surgical intervention:** definite and bridging treatment of NPWT  **Conservative treatment:** irrigation and close drainage | | | | | | | | | |
| --- | --- | --- | --- | --- | --- | --- | --- | --- | --- |
| **Outcome** | **Benefit or harm of intervention** | **Domains** | | | | | | | **Overall Risk of Bias** |
|  |  | **Bias due to confounding** | **Bias in selection of participants into the study** | **Bias in classification of interventions** | **Bias due to deviations from intended interventions** | **Bias due to missing data** | **Bias in measurement of outcomes** | **Bias in selection of the reported result** |  |
| **Mortality** | **Benefit** | **Moderate** | **Serious^1^** | **Moderate** | **Low** | **Moderate** | **Serious^2^** | **Moderate** | **Serious** |
| **Explanatory Footnotes**  1. Bias in selection of participants into the study because they were by surgeon’s decision instead of historical or pre-interventions  2. Bias due to measurement of outcomes because results of this study cannot be double blinded as an outcomes accessor | | | | | | | | | |

| **Title: Sjogren 2005**  **Type of study**: Retrospective  **Participants**: mediastinitis following open cardiac surgery  **Surgical intervention**: rewiring and closure after NPWT  **Conservative treatment**: rewiring, open dressing, closed irrigation, pectoralis flap and Omentoplasty | | | | | | | | | |
| --- | --- | --- | --- | --- | --- | --- | --- | --- | --- |
| **Outcome** | **Benefit or harm of intervention** | **Domains** | | | | | | | **Overall Risk of Bias** |
|  |  | **Bias due to confounding** | **Bias in selection of participants into the study** | **Bias in classification of interventions** | **Bias due to deviations from intended interventions** | **Bias due to missing data** | **Bias in measurement of outcomes** | **Bias in selection of the reported result** |  |
| **Mortality** | **Benefit** | **Moderate** | **Low** | **Moderate** | **Low** | **Moderate** | **Serious^1^** | **Moderate** | **Serious** |
| **Explanatory Footnotes**  1. Bias due to measurement outcomes because outcomes assessor was not clearly stated | | | | | | | | | |

| **Title: Chen 2008**  **Type of study:** Retrospective  **Participants:** deep sternal wound infection following open cardiac surgery  **Surgical intervention:** definite and bridging treatment of NPWT  **Conservative treatment:** delay closure and muscle flaps reconstruction | | | | | | | | | |
| --- | --- | --- | --- | --- | --- | --- | --- | --- | --- |
| **Outcome** | **Benefit or harm of intervention** | **Domains** | | | | | | | **Overall Risk of Bias** |
|  |  | **Bias due to confounding** | **Bias in selection of participants into the study** | **Bias in classification of interventions** | **Bias due to deviations from intended interventions** | **Bias due to missing data** | **Bias in measurement of outcomes** | **Bias in selection of the reported result** |  |
| **Mortality** | **Benefit** | **Serious^1^** | **Moderate** | **Moderate** | **Moderate** | **Low** | **Serious^2^** | **Low** | **Serious** |
| **Explanatory Footnotes**  1. Bias due to confounding because some potential confounders like age, sex, duration of therapy time, time from surgery, and different degree of infection involvement  2. Bias due to measurement of outcomes because results of this study cannot be double blinded as an outcomes accessor | | | | | | | | | |

| **Title: Eyileten 2009**  **Type of study:** Retrospective study  **Participants:** deep sternal wound infection following open cardiac surgery  **Surgical intervention:** definite and bridging treatment of NPWT  **Conservative treatment:** early bilateral pectoralis major muscle flap and continuous irrigation | | | | | | | | | |
| --- | --- | --- | --- | --- | --- | --- | --- | --- | --- |
| **Outcome** | **Benefit or harm of intervention** | **Domains** | | | | | | | **Overall Risk of Bias** |
|  |  | **Bias due to confounding** | **Bias in selection of participants into the study** | **Bias in classification of interventions** | **Bias due to deviations from intended interventions** | **Bias due to missing data** | **Bias in measurement of outcomes** | **Bias in selection of the reported result** |  |
| **Mortality** | **Benefit** | **Serious^1^** | **Serious^2^** | **Moderate** | **Low** | **Low** | **Serious^3^** | **Moderate** | **Serious** |
| **Explanatory Footnotes**  1. Bias due to confounding because some potential confounders like age, sex, duration of therapy time, time from surgery, and different degree of infection involvement  2. Bias in selection of participants into the study because they were by surgeon’s decision instead of historical or pre-interventions  3. Bias due to measurement of outcomes because results of this study cannot be double blinded as an outcomes accessor | | | | | | | | | |

| **Title:** **Baillot 2010**  **Type of study:** Retrospective study  **Participants:** deep sternal wound infection following open cardiac surgery  **Surgical intervention:** bridging treatment of NPWT to pectoralis muscle flap  **Conservative treatment:**debridement and drainage follow with pectoralis muscle flap | | | | | | | | | |
| --- | --- | --- | --- | --- | --- | --- | --- | --- | --- |
| **Outcome** | **Benefit or harm of intervention** | **Domains** | | | | | | | **Overall Risk of Bias** |
|  |  | **Bias due to confounding** | **Bias in selection of participants into the study** | **Bias in classification of interventions** | **Bias due to deviations from intended interventions** | **Bias due to missing data** | **Bias in measurement of outcomes** | **Bias in selection of the reported result** |  |
| **Mortality** | **Benefit** | **Serious^1^** | **Moderate** | **Moderate** | **Low** | **Moderate** | **Serious^2^** | **Moderate** | **Serious** |
| **Explanatory Footnotes**  1. Bias due to confounding because some potential confounders like age, sex, duration of therapy time, time from surgery, and different degree of infection involvement  2. Bias due to measurement of outcomes because results of this study cannot be double blinded as an outcomes accessor | | | | | | | | | |

| **Title: De Feo 2010**  **Type of study:** Retrospective study  **Participants:** deep sternal wound infection following open cardiac surgery  **Surgical intervention:** bridging treatment of NPWT to pectoralis muscle flap  **Conservative treatment:** Close irrigation w/ antibiotics | | | | | | | | | |
| --- | --- | --- | --- | --- | --- | --- | --- | --- | --- |
| **Outcome** | **Benefit or harm of intervention** | **Domains** | | | | | | | **Overall Risk of Bias** |
|  |  | **Bias due to confounding** | **Bias in selection of participants into the study** | **Bias in classification of interventions** | **Bias due to deviations from intended interventions** | **Bias due to missing data** | **Bias in measurement of outcomes** | **Bias in selection of the reported result** |  |
| **Mortality** | **Benefit** | **Serious^1^** | **Serious^2^** | **Moderate** | **Low** | **Low** | **Serious^3^** | **Moderate** | **Serious** |
| **Explanatory Footnotes**  1. Bias due to confounding because some potential confounders like age, sex, duration of therapy time, time from surgery, and different degree of infection involvement  2. Bias in selection of participants into the study because they were by surgeon’s decision instead of historical or pre-interventions  3. Bias due to measurement of outcomes because results of this study cannot be double blinded as an outcomes accessor | | | | | | | | | |

| **Title: Petzina 2010**  **Type of study:** Retrospective study  **Participants:** mediastinitis following open cardiac surgery  **Surgical intervention:** rewiring and closure after NPWT  **Conservative treatment:** Debridement, drainage, irrigation and transposition of the greater omentum | | | | | | | | | |
| --- | --- | --- | --- | --- | --- | --- | --- | --- | --- |
| **Outcome** | **Benefit or harm of intervention** | **Domains** | | | | | | | **Overall Risk of Bias** |
|  |  | **Bias due to confounding** | **Bias in selection of participants into the study** | **Bias in classification of interventions** | **Bias due to deviations from intended interventions** | **Bias due to missing data** | **Bias in measurement of outcomes** | **Bias in selection of the reported result** |  |
| **Mortality** | **Benefit** | **Serious^1^** | **Low** | **Moderate** | **Moderate** | **Moderate** | **Serious^2^** | **Moderate** | **Serious** |
| **Explanatory Footnotes**  1. Bias due to confounding because some potential confounders like age, sex, duration of therapy time, time from surgery, and different degree of infection involvement  2. Bias due to measurement of outcomes because results of this study cannot be double blinded as an outcomes accessor | | | | | | | | | |

| **Title: Assmann 2011**  **Type of study:** Retrospective study  **Participants:** deep sternal wound infection following open cardiac surgery  **Surgical intervention:** definite and bridging treatment of NPWT  **Conservative treatment:** Primary rewiring and disinfectant irrigation | | | | | | | | | |
| --- | --- | --- | --- | --- | --- | --- | --- | --- | --- |
| **Outcome** | **Benefit or harm of intervention** | **Domains** | | | | | | | **Overall Risk of Bias** |
|  |  | **Bias due to confounding** | **Bias in selection of participants into the study** | **Bias in classification of interventions** | **Bias due to deviations from intended interventions** | **Bias due to missing data** | **Bias in measurement of outcomes** | **Bias in selection of the reported result** |  |
| **Mortality** | **Benefit** | **Serious^1^** | **Serious^2^** | **Moderate** | **Low** | **Low** | **Serious^3^** | **Moderate** | **Serious** |
| **Explanatory Footnotes**  1. Bias due to confounding because some potential confounders like age, sex, duration of therapy time, time from surgery, and different degree of infection involvement  2. Bias in selection of participants into the study because they were by surgeon’s decision instead of historical or pre-interventions  3. Bias due to measurement of outcomes because results of this study cannot be double blinded as an outcomes accessor | | | | | | | | | |

| **Title: Marisa De Feo 2011**  **Type of study:** Retrospective study  **Participants:** post-sternotomy mediastinitis after cardiac surgery  **Surgical intervention:** definite and bridging treatment of NPWT  **Conservative treatment:** bilateral pectoralis major muscle flap after continuous irrigation | | | | | | | | | |
| --- | --- | --- | --- | --- | --- | --- | --- | --- | --- |
| **Outcome** | **Benefit or harm of intervention** | **Domains** | | | | | | | **Overall Risk of Bias** |
|  |  | **Bias due to confounding** | **Bias in selection of participants into the study** | **Bias in classification of interventions** | **Bias due to deviations from intended interventions** | **Bias due to missing data** | **Bias in measurement of outcomes** | **Bias in selection of the reported result** |  |
| **Mortality** | **Benefit** | **Serious^1^** | **Low** | **Moderate** | **Low** | **Moderate** | **Serious^2^** | **Moderate** | **Serious** |
| **Explanatory Footnotes**  1. Bias due to confounding because some potential confounders like age, sex, duration of therapy time, time from surgery, and different degree of infection involvement  2. Bias due to measurement of outcomes because results of this study cannot be double blinded as an outcomes accessor | | | | | | | | | |

| **Title:De Feo 2011**  **Type of study:** Retrospective study  **Participants:** deep sternal wound infection following open cardiac surgery  **Surgical intervention:** bridging treatment of NPWT to Pectoralis muscle flap reconstruction  **Conservative treatment:** debridement, irrigation and drainage follow with pectoralis muscle flap | | | | | | | | | |
| --- | --- | --- | --- | --- | --- | --- | --- | --- | --- |
| **Outcome** | **Benefit or harm of intervention** | **Domains** | | | | | | | **Overall Risk of Bias** |
|  |  | **Bias due to confounding** | **Bias in selection of participants into the study** | **Bias in classification of interventions** | **Bias due to deviations from intended interventions** | **Bias due to missing data** | **Bias in measurement of outcomes** | **Bias in selection of the reported result** |  |
| **Mortality** | **Benefit** | **Moderate** | **Moderate** | **Moderate** | **Moderate** | **Low** | **Serious^1^** | **Moderate** | **Serious** |
| **Explanatory Footnotes**  1. Bias due to measurement of outcomes because results of this study cannot be double blinded as an outcomes accessor | | | | | | | | | |

| **Title: Kobayashi 2011**  **Type of study:** Retrospective study  **Participants:** deep sternal wound infection following open cardiac surgery  **Surgical intervention:**bridging treatment of NPWT follow with omental flap transposition and pectoralis muscle flap reconstruction  **Conservative treatment:** debridement, irrigation and drainage follow with pectoralis muscle flap or omentum transposition | | | | | | | | | |
| --- | --- | --- | --- | --- | --- | --- | --- | --- | --- |
| **Outcome** | **Benefit or harm of intervention** | **Domains** | | | | | | | **Overall Risk of Bias** |
|  |  | **Bias due to confounding** | **Bias in selection of participants into the study** | **Bias in classification of interventions** | **Bias due to deviations from intended interventions** | **Bias due to missing data** | **Bias in measurement of outcomes** | **Bias in selection of the reported result** |  |
| **Mortality** | **Benefit** | **Moderate** | **Moderate** | **Moderate** | **Low** | **Moderate** | **Serious^1^** | **Moderate** | **Serious** |
| **Explanatory Footnotes**  1. Bias due to measurement of outcomes because results of this study cannot be double blinded as an outcomes accessor | | | | | | | | | |

| **Title: Morisaki 2011**  **Type of study:** Retrospective study  **Participants:** mediastinits following open cardiac surgery  **Surgical intervention:** bridging treatment of NPWT follow with omental flap transposition or pectoralis muscle flap reconstruction  **Conservative treatment:** simple closure or tissue flap reconstruction after debridement | | | | | | | | | |
| --- | --- | --- | --- | --- | --- | --- | --- | --- | --- |
| **Outcome** | **Benefit or harm of intervention** | **Domains** | | | | | | | **Overall Risk of Bias** |
|  |  | **Bias due to confounding** | **Bias in selection of participants into the study** | **Bias in classification of interventions** | **Bias due to deviations from intended interventions** | **Bias due to missing data** | **Bias in measurement of outcomes** | **Bias in selection of the reported result** |  |
| **Mortality** | **Benefit** | **Serious^1^** | **Low** | **Moderate** | **Serious^1^** | **Low** | **Serious^1^** | **Moderate** | **Serious** |
| **Explanatory Footnotes**  1. Bias due to confounding because some potential confounders like age, sex, duration of NPWT, underlying disease  2. Bias due to deviations from intended interventions because of non-determined closure method  3. Bias due to measurement of outcomes because results of this study cannot be double blinded as an outcomes accessor | | | | | | | | | |

| **Title: Deniz 2012**  **Type of study:** Retrospective study  **Participants:** Adults with DSWI  **Intervention:** Negative pressure wound therapy  **Conservative treatment:** surgical revision and debridement, open dressing, closed irrigation, pectoral flap | | | | | | | | | |
| --- | --- | --- | --- | --- | --- | --- | --- | --- | --- |
| **Outcome** | **Benefit or harm of intervention** | **Domains** | | | | | | | **Overall Risk of Bias** |
|  |  | **Bias due to confounding** | **Bias in selection of participants into the study** | **Bias in classification of interventions** | **Bias due to deviations from intended interventions** | **Bias due to missing data** | **Bias in measurement of outcomes** | **Bias in selection of the reported result** |  |
| **Mortality** | **Benefit** | **Serious^1^** | **Moderate** | **Moderate^2^** | **Low** | **Serious^3^** | **Serious^4^** | **Low** | **Serious** |
| **Explanatory Footnotes**  1. Bias due to confounding because some potential confounders like age, sex, duration of NPWT, underlying disease  2. Bias due to classification of interventions because data information extracted from medical records  3. Bias due to missing data because information about missing data for main outcome was not described  4. Bias due to measurement of outcomes because outcomes assessor was not clearly stated | | | | | | | | | |

| **Title: Rodriguez CB 2012**  **Type of study:** Retrospective study  **Participants:** Adults with DSWI  **Intervention:** Negative pressure wound therapy  **Conservative treatment:** Direct wound closure (debride, wound irrigation, rewiring) | | | | | | | | | |
| --- | --- | --- | --- | --- | --- | --- | --- | --- | --- |
| **Outcome** | **Benefit or harm of intervention** | **Domains** | | | | | | | **Overall Risk of Bias** |
|  |  | **Bias due to confounding** | **Bias in selection of participants into the study** | **Bias in classification of interventions** | **Bias due to deviations from intended interventions** | **Bias due to missing data** | **Bias in measurement of outcomes** | **Bias in selection of the reported result** |  |
| **Mortality** | **Benefit** | **Serious^1^** | **Moderate** | **Moderate^2^** | **Low** | **Serious^3^** | **Serious^4^** | **Low** | **Serious** |
| **Explanatory Footnotes**  1. Bias due to confounding because some potential confounders like age, sex, duration of NPWT, underlying disease  2. Bias due to classification of interventions because data information extracted from medical records  3. Bias due to missing data because information about missing data for main outcome was not described  4. Bias due to measurement of outcomes because outcomes assessor was not clearly stated | | | | | | | | | |

| **Title: Simek 2012**  **Type of study:** Retrospective study  **Participants:** Adults with DSWI  **Intervention:** Negative pressure wound therapy  **Conservative treatment:** close irrigation | | | | | | | | | |
| --- | --- | --- | --- | --- | --- | --- | --- | --- | --- |
| **Outcome** | **Benefit or harm of intervention** | **Domains** | | | | | | | **Overall Risk of Bias** |
|  |  | **Bias due to confounding** | **Bias in selection of participants into the study** | **Bias in classification of interventions** | **Bias due to deviations from intended interventions** | **Bias due to missing data** | **Bias in measurement of outcomes** | **Bias in selection of the reported result** |  |
| **Mortality** | **Benefit** | **Serious^1^** | **Serious^2^** | **Moderate** | **Moderate** | **Moderate** | **Serious^3^** | **Moderate** | **Serious** |
| **Explanatory Footnotes**  1. Bias due to confounding because some potential confounders like age, sex, duration of NPWT, underlying disease  2. Bias in selection of participants into the study because choices were made by surgeon’s decision instead of historical or pre-interventions  3. Bias due to measurement of outcomes because outcomes assessor was not clearly stated | | | | | | | | | |

| **Title: Steingrimsson 2012**  **Type of study:** Retrospective study  **Participants:** Adults with DSWI  **Intervention:** Negative pressure wound therapy  **Conservative treatment:** Open and/or close irrigation | | | | | | | | | |
| --- | --- | --- | --- | --- | --- | --- | --- | --- | --- |
| **Outcome** | **Benefit or harm of intervention** | **Domains** | | | | | | | **Overall Risk of Bias** |
|  |  | **Bias due to confounding** | **Bias in selection of participants into the study** | **Bias in classification of interventions** | **Bias due to deviations from intended interventions** | **Bias due to missing data** | **Bias in measurement of outcomes** | **Bias in selection of the reported result** |  |
| **Mortality** | **Benefit** | **Serious^1^** | **Moderate** | **Serious^2^** | **Low** | **Moderate** | **Serious^3^** | **Moderate** | **Serious** |
| **Explanatory Footnotes**  1. Bias due to confounding because some potential confounders like age, sex, duration of NPWT, underlying disease  2. Bias due to classification of interventions because data information extracted from medical records  3. Bias due to measurement of outcomes because outcomes assessor was not clearly stated | | | | | | | | | |

| **Title: Vos RJ 2012**  **Type of study:** Retrospective study  **Participants:** Adults with DSWI  **Intervention:** Negative pressure wound therapy  **Conservative treatment:** Open packing | | | | | | | | | |
| --- | --- | --- | --- | --- | --- | --- | --- | --- | --- |
| **Outcome** | **Benefit or harm of intervention** | **Domains** | | | | | | | **Overall Risk of Bias** |
|  |  | **Bias due to confounding** | **Bias in selection of participants into the study** | **Bias in classification of interventions** | **Bias due to deviations from intended interventions** | **Bias due to missing data** | **Bias in measurement of outcomes** | **Bias in selection of the reported result** |  |
| **Mortality** | **Benefit** | **Serious^1^** | **Serious^2^** | **Moderate^3^** | **Moderate** | **Low** | **Serious^4^** | **Low** | **Serious** |
| **Explanatory Footnotes**  1. Bias due to confounding because some potential confounders like age, sex, duration of NPWT, underlying disease  2. Bias in selection of participants into the study because choices were made by surgeon’s decision instead of historical or pre-interventions  3. Bias due to classification of interventions because data information extracted from medical records  4. Bias due to measurement of outcomes because outcomes assessor was not clearly stated | | | | | | | | | |

| **Title: Vos 2012**  **Type of study:** Retrospective study  **Participants:** Adults with DSWI  **Intervention:** Negative pressure wound therapy  **Conservative treatment:** Close drainage with Redon catheter | | | | | | | | | |
| --- | --- | --- | --- | --- | --- | --- | --- | --- | --- |
| **Outcome** | **Benefit or harm of intervention** | **Domains** | | | | | | | **Overall Risk of Bias** |
|  |  | **Bias due to confounding** | **Bias in selection of participants into the study** | **Bias in classification of interventions** | **Bias due to deviations from intended interventions** | **Bias due to missing data** | **Bias in measurement of outcomes** | **Bias in selection of the reported result** |  |
| **Mortality** | **Benefit** | **Serious^1^** | **Serious^2^** | **Moderate^3^** | **Low** | **Moderate** | **Serious^4^** | **Low** | **Serious** |
| **Explanatory Footnotes**  1. Bias due to confounding because some potential confounders like age, sex, duration of NPWT, underlying disease  2. Bias in selection of participants into the study because choices were made by surgeon’s decision instead of historical or pre-interventions  3. Bias due to classification of interventions because data information extracted from medical records  4. Bias due to measurement of outcomes because outcomes assessor was not clearly stated | | | | | | | | | |

| **Title: Fleck T 2014**  **Type of study:** Retrospective study  **Participants:** Adults with DSWI  **Intervention:** Negative pressure wound therapy  **Conservative treatment:** Surgical debridement, irrigation, open packing, muscle flap | | | | | | | | | |
| --- | --- | --- | --- | --- | --- | --- | --- | --- | --- |
| **Outcome** | **Benefit or harm of intervention** | **Domains** | | | | | | | **Overall Risk of Bias** |
|  |  | **Bias due to confounding** | **Bias in selection of participants into the study** | **Bias in classification of interventions** | **Bias due to deviations from intended interventions** | **Bias due to missing data** | **Bias in measurement of outcomes** | **Bias in selection of the reported result** |  |
| **Mortality** | **Benefit** | **Serious^1^** | **Serious^2^** | **Moderate^3^** | **Moderate** | **Low** | **Serious^4^** | **Moderate** | **Serious** |
| **Explanatory Footnotes**  1. Bias due to confounding because some potential confounders like age, sex, duration of NPWT, underlying disease  2. Bias in selection of participants into the study because choices were made by surgeon’s decision instead of historical or pre-interventions  3. Bias due to classification of interventions because data information extracted from medical records  4. Bias due to measurement of outcomes because outcomes assessor was not clearly stated | | | | | | | | | |

| **Title: Risnes I 2014**  **Type of study:** Retrospective study  **Participants:** Adults with DSWI  **Intervention:** Negative pressure wound therapy  **Conservative treatment:** Traditional close drainage with irrigation | | | | | | | | | |
| --- | --- | --- | --- | --- | --- | --- | --- | --- | --- |
| **Outcome** | **Benefit or harm of intervention** | **Domains** | | | | | | | **Overall Risk of Bias** |
|  |  | **Bias due to confounding** | **Bias in selection of participants into the study** | **Bias in classification of interventions** | **Bias due to deviations from intended interventions** | **Bias due to missing data** | **Bias in measurement of outcomes** | **Bias in selection of the reported result** |  |
| **Mortality** | **Benefit** | **Moderate^1^** | **Serious^2^** | **Moderate^3^** | **Low** | **Moderate** | **Serious^4^** | **Moderate** | **Serious** |
| **Explanatory Footnotes**  1. Bias due to confounding because some potential confounders like age, sex, duration of NPWT, underlying disease  2. Bias in selection of participants into the study because choices were made by surgeon’s decision instead of historical or pre-interventions  3. Bias due to classification of interventions because data information extracted from medical records  4. Bias due to measurement of outcomes because outcomes assessor was not clearly stated | | | | | | | | | |

| **Title: Yumun G 2014**  **Type of study:** Retrospective study  **Participants:** Adults with DSWI  **Intervention:** Negative pressure wound therapy  **Conservative treatment:** Primary surgical closure | | | | | | | | | |
| --- | --- | --- | --- | --- | --- | --- | --- | --- | --- |
| **Outcome** | **Benefit or harm of intervention** | **Domains** | | | | | | | **Overall Risk of Bias** |
|  |  | **Bias due to confounding** | **Bias in selection of participants into the study** | **Bias in classification of interventions** | **Bias due to deviations from intended interventions** | **Bias due to missing data** | **Bias in measurement of outcomes** | **Bias in selection of the reported result** |  |
| **Mortality** | **Benefit** | **Moderate^1^** | **Moderate** | **Serious^2^** | **Low** | **Moderate** | **Serious^3^** | **Low** | **Serious** |
| **Explanatory Footnotes**  1. Bias due to confounding because some potential confounders like age, sex, duration of NPWT, underlying disease  2. Bias due to classification of interventions because data information extracted from medical records  3. Bias due to measurement of outcomes because outcomes assessor was not clearly stated | | | | | | | | | |

| **Title: Morisaki 2016**  **Type of study:** Retrospective study  **Participants:** Adults with DSWI  **Intervention:** Negative pressure wound therapy  **Conservative treatment:** Open daily irrigation | | | | | | | | | |
| --- | --- | --- | --- | --- | --- | --- | --- | --- | --- |
| **Outcome** | **Benefit or harm of intervention** | **Domains** | | | | | | | **Overall Risk of Bias** |
|  |  | **Bias due to confounding** | **Bias in selection of participants into the study** | **Bias in classification of interventions** | **Bias due to deviations from intended interventions** | **Bias due to missing data** | **Bias in measurement of outcomes** | **Bias in selection of the reported result** |  |
| **Mortality** | **Benefit** | **Moderate^1^** | **Low** | **Serious^2^** | **Low** | **Moderate** | **Serious^3^** | **Moderate** | **Serious** |
| **Explanatory Footnotes**  1. Bias due to confounding because some potential confounders like age, sex, duration of NPWT, underlying disease  2. Bias due to classification of interventions because data information extracted from medical records  3. Bias due to measurement of outcomes because outcomes assessor was not clearly stated | | | | | | | | | |

| **Title: Pan T 2020**  **Type of study:** Retrospective study  **Participants:** Adults with DSWI  **Intervention:** Negative pressure wound therapy  **Conservative treatment:** Open daily irrigation | | | | | | | | | |
| --- | --- | --- | --- | --- | --- | --- | --- | --- | --- |
| **Outcome** | **Benefit or harm of intervention** | **Domains** | | | | | | | **Overall Risk of Bias** |
|  |  | **Bias due to confounding** | **Bias in selection of participants into the study** | **Bias in classification of interventions** | **Bias due to deviations from intended interventions** | **Bias due to missing data** | **Bias in measurement of outcomes** | **Bias in selection of the reported result** |  |
| **Mortality** | **Benefit** | **Serious^1^** | **Serious^2^** | **Moderate^3^** | **Low** | **Moderate** | **Serious^4^** | **Low** | **Serious** |
| **Explanatory Footnotes**  1. Bias due to confounding because some potential confounders like age, sex, duration of NPWT, underlying disease  2. Bias in selection of participants into the study because choices were made by surgeon’s decision instead of historical or pre-interventions  3. Bias due to classification of interventions because data information extracted from medical records  4. Bias due to measurement of outcomes because outcomes assessor was not clearly stated | | | | | | | | | |

| **Title: Hämäläinen E 2021**  **Type of study:** Retrospective study  **Participants:** Adults with DSWI  **Intervention:** Negative pressure wound therapy  **Conservative treatment:** Open daily irrigation | | | | | | | | | |
| --- | --- | --- | --- | --- | --- | --- | --- | --- | --- |
| **Outcome** | **Benefit or harm of intervention** | **Domains** | | | | | | | **Overall Risk of Bias** |
|  |  | **Bias due to confounding** | **Bias in selection of participants into the study** | **Bias in classification of interventions** | **Bias due to deviations from intended interventions** | **Bias due to missing data** | **Bias in measurement of outcomes** | **Bias in selection of the reported result** |  |
| **Mortality** | **Benefit** | **Serious^1^** | **Low** | **Serious^2^** | **Moderate** | **Low** | **Serious^3^** | **Moderate** | **Serious** |
| **Explanatory Footnotes**  1. Bias due to confounding because some potential confounders like age, sex, duration of NPWT, underlying disease  2. Bias due to classification of interventions because data information extracted from medical records  3. Bias due to measurement of outcomes because outcomes assessor was not clearly stated | | | | | | | | | |

| **Title: Myllykangas HM 2021**  **Type of study:** Retrospective study  **Participants:** Adults with DSWI  **Intervention:** Negative pressure wound therapy  **Conservative treatment:** Open daily irrigation | | | | | | | | | |
| --- | --- | --- | --- | --- | --- | --- | --- | --- | --- |
| **Outcome** | **Benefit or harm of intervention** | **Domains** | | | | | | | **Overall Risk of Bias** |
|  |  | **Bias due to confounding** | **Bias in selection of participants into the study** | **Bias in classification of interventions** | **Bias due to deviations from intended interventions** | **Bias due to missing data** | **Bias in measurement of outcomes** | **Bias in selection of the reported result** |  |
| **Mortality** | **Benefit** | **Moderate^1^** | **Serious^2^** | **Serious^3^** | **Low** | **Low** | **Serious^4^** | **Moderate** | **Serious** |
| **Explanatory Footnotes**  1. Bias due to confounding because some potential confounders like age, sex, duration of NPWT, underlying disease  2. Bias in selection of participants into the study because choices were made by surgeon’s decision instead of historical or pre-interventions  3. Bias due to classification of interventions because data information extracted from medical records  4. Bias due to measurement of outcomes because outcomes assessor was not clearly stated | | | | | | | | | |

| **Title: Banjanovic, B/ 2022**  **Type of study:** Retrospective study  **Participants:** Adults with DSWI  **Intervention:** Negative pressure wound therapy  **Conservative treatment:** Open daily irrigation | | | | | | | | | |
| --- | --- | --- | --- | --- | --- | --- | --- | --- | --- |
| **Outcome** | **Benefit or harm of intervention** | **Domains** | | | | | | | **Overall Risk of Bias** |
|  |  | **Bias due to confounding** | **Bias in selection of participants into the study** | **Bias in classification of interventions** | **Bias due to deviations from intended interventions** | **Bias due to missing data** | **Bias in measurement of outcomes** | **Bias in selection of the reported result** |  |
| **Mortality** | **Benefit** | **Moderate^1^** | **Serious^2^** | **Serious^3^** | **Moderate** | **Low** | **Serious^4^** | **Moderate** | **Serious** |
| **Explanatory Footnotes**  1. Bias due to confounding because some potential confounders like age, sex, duration of NPWT, underlying disease  2. Bias in selection of participants into the study because choices were made by surgeon’s decision instead of historical or pre-interventions  3. Bias due to classification of interventions because data information extracted from medical records  4. Bias due to measurement of outcomes because outcomes assessor was not clearly stated | | | | | | | | | |

| **Title: Gegouskov V 2022**  **Type of study:** Retrospective study  **Participants:** Adults with DSWI  **Intervention:** Negative pressure wound therapy  **Conservative treatment:** Open daily irrigation | | | | | | | | | |
| --- | --- | --- | --- | --- | --- | --- | --- | --- | --- |
| **Outcome** | **Benefit or harm of intervention** | **Domains** | | | | | | | **Overall Risk of Bias** |
|  |  | **Bias due to confounding** | **Bias in selection of participants into the study** | **Bias in classification of interventions** | **Bias due to deviations from intended interventions** | **Bias due to missing data** | **Bias in measurement of outcomes** | **Bias in selection of the reported result** |  |
| **Mortality** | **Benefit** | **Serious^1^** | **Low** | **Serious^2^** | **Moderate** | **Moderate** | **Serious^3^** | **Low** | **Serious** |
| **Explanatory Footnotes**  1. Bias due to confounding because some potential confounders like age, sex, duration of NPWT, underlying disease  2. Bias due to classification of interventions because data information extracted from medical records  3. Bias due to measurement of outcomes because outcomes assessor was not clearly stated | | | | | | | | | |

| **Title: Myllykangas HM 2022**  **Type of study:** Retrospective study  **Participants:** Adults with DSWI  **Intervention:** Negative pressure wound therapy  **Conservative treatment:** Open daily irrigation | | | | | | | | | |
| --- | --- | --- | --- | --- | --- | --- | --- | --- | --- |
| **Outcome** | **Benefit or harm of intervention** | **Domains** | | | | | | | **Overall Risk of Bias** |
|  |  | **Bias due to confounding** | **Bias in selection of participants into the study** | **Bias in classification of interventions** | **Bias due to deviations from intended interventions** | **Bias due to missing data** | **Bias in measurement of outcomes** | **Bias in selection of the reported result** |  |
| **Mortality** | **Benefit** | **Serious^1^** | **Serious^2^** | **Moderate** | **Low** | **Low** | **Serious^3^** | **Moderate** | **Serious** |
| **Explanatory Footnotes**  1. Bias due to confounding because some potential confounders like age, sex, duration of NPWT, underlying disease  2. Bias in selection of participants into the study because choices were made by surgeon’s decision instead of historical or pre-interventions  3. Bias due to measurement of outcomes because outcomes assessor was not clearly stated | | | | | | | | | |

| **Title: Akbayrak H 2023**  **Type of study:** Retrospective study  **Participants:** Adults with DSWI  **Intervention:** Negative pressure wound therapy  **Conservative treatment:** Open daily irrigation | | | | | | | | | |
| --- | --- | --- | --- | --- | --- | --- | --- | --- | --- |
| **Outcome** | **Benefit or harm of intervention** | **Domains** | | | | | | | **Overall Risk of Bias** |
|  |  | **Bias due to confounding** | **Bias in selection of participants into the study** | **Bias in classification of interventions** | **Bias due to deviations from intended interventions** | **Bias due to missing data** | **Bias in measurement of outcomes** | **Bias in selection of the reported result** |  |
| **Mortality** | **Benefit** | **Serious^1^** | **Moderate** | **Moderate** | **Serious^2^** | **Low** | **Serious^3^** | **Moderate** | **Serious** |
| **Explanatory Footnotes**  1. Bias due to confounding because some potential confounders like age, sex, duration of therapy time, time from surgery, and different degree of infection involvement  2. Bias due to deviations from intended interventions because of non-determined closure method  3. Bias due to measurement of outcomes because results of this study cannot be double blinded for the outcomes accessor | | | | | | | | | |

| **Title: Saltarocchi S 2023**  **Type of study:** Retrospective study  **Participants:** Adults with DSWI  **Intervention:** Negative pressure wound therapy  **Conservative treatment:** Open daily irrigation | | | | | | | | | |
| --- | --- | --- | --- | --- | --- | --- | --- | --- | --- |
| **Outcome** | **Benefit or harm of intervention** | **Domains** | | | | | | | **Overall Risk of Bias** |
|  |  | **Bias due to confounding** | **Bias in selection of participants into the study** | **Bias in classification of interventions** | **Bias due to deviations from intended interventions** | **Bias due to missing data** | **Bias in measurement of outcomes** | **Bias in selection of the reported result** |  |
| **Mortality** | **Benefit** | **Serious^1^** | **Serious^2^** | **Moderate** | **Low** | **Low** | **Serious^3^** | **Moderate** | **Serious** |
| **Explanatory Footnotes**  1. Bias due to confounding because some potential confounders like age, sex, duration of NPWT, underlying disease  2. Bias in selection of participants into the study because choices were made by surgeon’s decision instead of historical or pre-interventions  3. Bias due to measurement of outcomes because outcomes assessor was not clearly stated | | | | | | | | | |

| Risk of Bias of Non Randomized Controlled Trials | | | | | | | | |
| --- | --- | --- | --- | --- | --- | --- | --- | --- |
| Study | **ROBINS-I Domains** | | | | | | | |
|  | **D1** | **D2** | **D3** | **D4** | **D5** | **D6** | **D7** | **Overall** |
| Berg et al 2000 |  |  |  |  |  |  |  |  |
| Doss et al 2002 |  |  |  |  |  |  |  |  |
| Fleck TM et al 2002 |  |  |  |  |  |  |  |  |
| Domkowski et al 2003 |  |  |  |  |  |  |  |  |
| Fuchs et al 2005 |  |  |  |  |  |  |  |  |
| Immer et al 2005 |  |  |  |  |  |  |  |  |
| Segers et al 2005 |  |  |  |  |  |  |  |  |
| Sjogren et al 2005 |  |  |  |  |  |  |  |  |
| Chen Y et al 2008 |  |  |  |  |  |  |  |  |
| Eyileten et al 2009 |  |  |  |  |  |  |  |  |
| Baillot, R et al 2010 |  |  |  |  |  |  |  |  |
| De Feo et al 2010 |  |  |  |  |  |  |  |  |
| Petzina et al 2010 |  |  |  |  |  |  |  |  |
| Assmann et al 2011 |  |  |  |  |  |  |  |  |
| Marisa De Feo et al 2011 |  |  |  |  |  |  |  |  |
| De Feo et al 2011 |  |  |  |  |  |  |  |  |
| Kobayashi et al 2011 |  |  |  |  |  |  |  |  |
| Morisaki et al 2011 |  |  |  |  |  |  |  |  |
| Deniz et al 2012 |  |  |  |  |  |  |  |  |
| Rodriguez CB et al 2012 |  |  |  |  |  |  |  |  |
| Simek et al 2012 |  |  |  |  |  |  |  |  |
| Steingrimsson et al 2012 |  |  |  |  |  |  |  |  |
| Vos RJ et al 2012 |  |  |  |  |  |  |  |  |
| Vos et al 2012 |  |  |  |  |  |  |  |  |
| Fleck T et al 2014 |  |  |  |  |  |  |  |  |
| Risnes I et al 2014 |  |  |  |  |  |  |  |  |
| Yumun G et al 2014 |  |  |  |  |  |  |  |  |
| Morisaki et al 2016 |  |  |  |  |  |  |  |  |
| Pan T et al 2020 |  |  |  |  |  |  |  |  |
| Hämäläinen E et al 2021 |  |  |  |  |  |  |  |  |
| Myllykangas HM et al 2021 |  |  |  |  |  |  |  |  |
| Banjanovic, B et al 2022 |  |  |  |  |  |  |  |  |
| Gegouskov V et al 2022 |  |  |  |  |  |  |  |  |
| Myllykangas, HM et al 2022 |  |  |  |  |  |  |  |  |
| Akbayrak, H et al 2023 |  |  |  |  |  |  |  |  |
| Saltarocchi, S et al 2023 |  |  |  |  |  |  |  |  |
| D1: Bias due to confounding  D2: Bias in selection of participants  D3: Bias in classification of interventions  D4: Bias due to deviations from intended interventions  D5: Bias due to missing data  D6: Bias in measurement of outcomes  D7: Bias in selection of reported results  Green: Low risk; Yellow: moderate risk; Orange: serious risk | | | | | | | | |
