# Supplementary material for: Effectiveness and safety of negative pressure wound therapy in patients with deep sternal wound infection: a systematic review and meta-analysis
Source: Int J Surg. 2024 Nov 14;110(12):8107–25. doi: 10.1097/JS9.0000000000002138 (PMC11634157; doi:10.1097/JS9.0000000000002138)
Supplement: SUPPLEMENTARY MATERIAL [file js9-110-8107-s006.docx]

| First Author  /Year | Wound healing time  (Mean ± SD, days)  (NPWT vs conventional) | Description of wound healing |
| --- | --- | --- |
| Doss  2002 | 17.2 (±5.8) vs. 22.9 (±10.8) | Secondary wound healing showed an average of 3.2 cm^2^/day reduction in wound size in the conventional group and 4.63 cm^2^/day in the vacuum-assisted suction drainage group. |
| Fuchs  2005 | 20.75 (±8.51) vs 33.33 (±27.86) | Until the sternum was rewired (primary wound rewiring), and wound healing was achieved without rewiring (secondary wound healing). |
| Segers  2005 | Not mentioned | 28 patients (44.4%) complete healing was not achieved at discharge and prolonged treatment as an outpatient was necessary. |
| Chen Y  2008 | Not mentioned | The wound healing time cannot be determined accurately. Many of the vacuum assisted closure patients were discharged to the care of the Royal District Nursing Service. |
| De Feo  2010 | 13.5 (±3.2) vs 21.2 (±16.4) | If the cultures were sterile, and wound healing was satisfactory, the patient was discharged. |
| Kobayashi  2011 | 63.4 (±54.1) vs 120.0 (±31.8) | Duration of treatment were calculated after the onset of infection to the day of healing according to surgeon’s judgement. |
| Akbayrak H  2023 | 20.63 (±8.87) vs 56.41 (±28.5) | The treatment was completed by sternal rewiring or secondary healing. |
